# Cryo‐EM Analysis of a Tri‐Heme Cytochrome‐Associated RC‐LH1 Complex from the Marine Photoheterotrophic Bacterium *Dinoroseobacter Shibae*

**DOI:** 10.1002/advs.202413456

**Published:** 2025-03-20

**Authors:** Weiwei Wang, Yanting Liu, Jiayi Gu, Shaoya An, Cheng Ma, Haichun Gao, Nianzhi Jiao, Jian‐Ren Shen, John Thomas Beatty, Michal Koblížek, Xing Zhang, Qiang Zheng, Jing‐Hua Chen

**Affiliations:** ^1^ College of Life Sciences Zhejiang University Hangzhou Zhejiang 310058 China; ^2^ State Key Laboratory of Marine Environmental Science College of Ocean and Earth Sciences Xiamen University Xiamen Fujian 361005 China; ^3^ Department of Pathology of Sir Run Run Shaw Hospital Department of Biophysics Zhejiang University School of Medicine Hangzhou Zhejiang 310058 China; ^4^ Center of Cryo Electron Microscopy Zhejiang University School of Medicine Hangzhou Zhejiang 310058 China; ^5^ Photosynthesis Research Center Key Laboratory of Photobiology Institute of Botany Chinese Academy of Sciences Beijing 100093 China; ^6^ Research Institute for Interdisciplinary Science and Graduate School of Natural Science and Technology Okayama University Okayama 700–8530 Japan; ^7^ Department of Microbiology & Immunology University of British Columbia Vancouver BC V6T 1Z3 Canada; ^8^ Laboratory of Anoxygenic Phototrophs Institute of Microbiology Czech Academy of Science Třeboň 37981 Czechia

**Keywords:** energy transfer, photoheterotrophic bacteria, photosynthesis, reaction center, structure

## Abstract

The reaction center‐light harvesting 1 (RC‐LH1) complex converts solar energy into electrical energy, driving the initiation of photosynthesis. The authors present a cryo‐electron microscopy structure of the RC‐LH1 isolated from a marine photoheterotrophic bacterium *Dinoroseobacter shibae*. The RC comprises four subunits, including a three‐heme cytochrome (Cyt) *c* protein, and is surrounded by a closed LH ring composed of 17 pairs of antenna subunits. Notably, a novel subunit with an N‐terminal “helix‐turn‐helix” motif embedded in the gap between the RC and the LH ring is identified. The purified RC‐LH1 complex exhibits high stability in solutions containing Mg^2+^ or Ca^2+^. The periplasmic Cyt *c*_2_ is predicted to bind at the junction between the Cyt subunit and the membrane plane, enabling electron transfer from Cyt *c*_2_ to the proximal heme of the tri‐heme Cyt, and subsequently to the special pair of bacteriochlorophylls. These findings provide structural insights into the efficient energy and electron transfer processes within a distinct type of RC‐LH1, and shed light on evolutionary adaptations of photosynthesis.

## Graphical abstract

Cryo‐EM reveals a closed 17‐subunit LH ring encircling the RC‐LH1 complex in *Dinoroseobacter shibae*, featuring a novel helix‐turn‐helix subunit bridging RC and LH. Mg^2^⁺/Ca^2^⁺ stabilizes the complex, while periplasmic Cyt *c*₂ mediates electron transfer via a tri‐heme Cyt to the special pair of bacteriochlorophylls. Insights into energy/electron efficiency and photosynthetic evolution are provided.

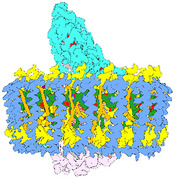

## 1 Introduction

Photosynthesis represents one of the most fundamental biological processes providing food and energy for most of the life forms on Earth. The initial steps of photosynthesis, light absorption, primary charge separation, and electron transfer, are conducted by special membrane‐embedded pigment‐protein complexes. Anoxygenic phototrophs such as purple bacteria or green non‐sulfur bacteria employ reaction center‐light harvesting 1 (RC‐LH1) complexes.^[^ ^1^ ^]^ LH1 absorbs sunlight and transfers the energy to the RC where a special pair of bacteriochlorophylls (SP) undergo excitation, leading to charge separation where electrons are transferred across the membrane through a series of cofactors within the RC, resulting in the reduction of a quinone electron acceptor and formation of a quinol through addition of protons derived from the cytoplasm.^[^ ^2^ ^]^ Subsequently, the quinols pass on the electrons to the cytochrome (Cyt) *bc*_1_ complex and return to the RC as quinones, resulting in the release of protons to the opposite (periplasmic) side of the membrane;^[^ ^3^ ^]^ the electrons of the Cyt *bc*_1_ complex are transferred back to the RC through periplasmic electron carriers, forming a cyclic electron transport chain. This process establishes a transmembrane proton gradient that drives the synthesis of adenosine triphosphate (ATP), providing the energy required for cellular metabolism.^[^ ^4^ ^]^

Given its important role, extensive research has been carried out on the RC‐LH1, with a focus on determining various RC‐LH1 structures.^[^ ^5^ ^]^ Generally, these RC‐LH1s can be classified as members of group‐II, which possesses a Cyt subunit containing four *c*‐type hemes, or group‐I which lacks a Cyt subunit, each with distinct cyclic electron transfer processes.^[^ ^6^ ^]^ In the case of RC‐LH1 with a bound Cyt *c* subunit, the photo‐oxidized SP is rapidly re‐reduced by one of the hemes in the subunit.^[^ ^6^ ^]^ In contrast, in the absence of a tightly bound Cyt subunit, soluble Cyt *c*_2_ serves as an immediate electron donor to the SP.^[^ ^7^ ^]^ It has been suggested that RC‐LH1s belonging to group‐I evolved from an ancestor with a tetraheme Cyt subunit, which was subsequently lost during evolution to generate the RC‐LH1 without the bound Cyt subunit.^[^ ^8^ ^]^ However, the structure and cyclic electron transfer mechanism of three‐heme Cyt subunit‐associated RC‐LH1s remain to be fully elucidated.

The bacterium *Dinoroseobacter shibae* (*D. shibae*) is a representative of the ecologically important *Roseobacter* clade, which plays a crucial role in the marine carbon cycle.^[^ ^9^ ^]^ *D. shibae* is phylogenetically related to the purple non‐sulfur bacterium *Rhodobacter* (*Rba*.) *sphaeroides* (order *Rhodobacteriales*). However, it is an aerobic anoxygenic photoheterotrophic bacterium (AAPB), which utilizes light energy to supplement its mostly heterotrophic metabolism.^[^ ^10^ ^]^ As it lacks inorganic carbon fixation, it represents an evolutionary transitional stage between photoautotrophs and chemoheterotrophs.^[^ ^11^ ^]^ The chromosome of *D. shibae* contains a complete photosynthetic gene cluster (PGC) which includes genes related to bacteriochlorophyll (BChl) *a* biosynthesis, carotenoid biosynthesis, photosynthetic apparatus assembly, and regulatory elements (Figure S1, Supporting Information).^[^ ^12^ ^]^ Notably, the photosynthetic system of *D. shibae* features a type‐II RC which shows absorption spectra in the infrared region similar to those of the purple bacterial RC‐LH1s.^[^ ^13^ ^]^ Bioinformatics analysis suggests that *D. shibae* RC contains a three‐heme Cyt *c* subunit and performs cyclic electron transport by a mechanism that is not yet fully understood.^[^ ^14^ ^]^

In this study, we isolated the RC‐LH1 complex from a representative *D. shibae* strain, DFL 12^T^,^[^ ^13^ ^]^ and found that the purified RC‐LH1 complex exhibited high stability in solutions containing magnesium (Mg^2+^) or calcium (Ca^2+^) ions. By using cryogenic electron microscopy (cryo‐EM), we determined three structures of the *Ds*RC‐LH1s purified in salt‐free, Mg^2+^‐containing, and EDTA‐2Na‐containing solutions with global resolutions of 2.7, 2.7, and 2.8 Å, respectively (Table S1, Supporting Information). These structures revealed the protein conformation and cofactor distribution within the *D. shibae* RC‐LH1 complex (*Ds*RC‐LH1), providing a structural basis for the efficient energy absorption and quenching mechanism of *Ds*RC‐LH1, and the possible environmental adaptation of AAPB during evolution.

## 2 Results

### 2.1 Overall Structure of the *Ds*RC‐LH1

The *Ds*RC‐LH1 purified using Mg^2+^ solutions shows high homogeneity with a sharp peak after gel filtration using a Superose 6 Increase column (Figure S2A,B, Supporting Information). Based on SDS‐PAGE analysis, we identified four RC subunits (L, M, H, and C) and two LH1 subunits (α and β) (Figure S2C, Supporting Information). Each subunit was further verified using liquid chromatography‐tandem mass spectrometry (LC‐MS/MS) (Table S2, Supporting Information). The *Ds*RC‐LH1 exhibits an absorption maximum at 867 nm, and a broad region of absorption in the range of 450–550 nm that corresponds to the bound carotenoids (Figure S2D, Supporting Information).^[^ ^13^ ^]^ The vitrified *Ds*RC‐LH1 sample (Table S2, Supporting Information) was imaged, and, using the single‐particle analysis approach the structure of the *Ds*RC‐LH1 was determined at a global resolution of 2.7 Å, as assessed by the gold standard Fourier shell correlation (FSC) at 0.143 (Figure S3, Supporting Information).

**Figure 1:**
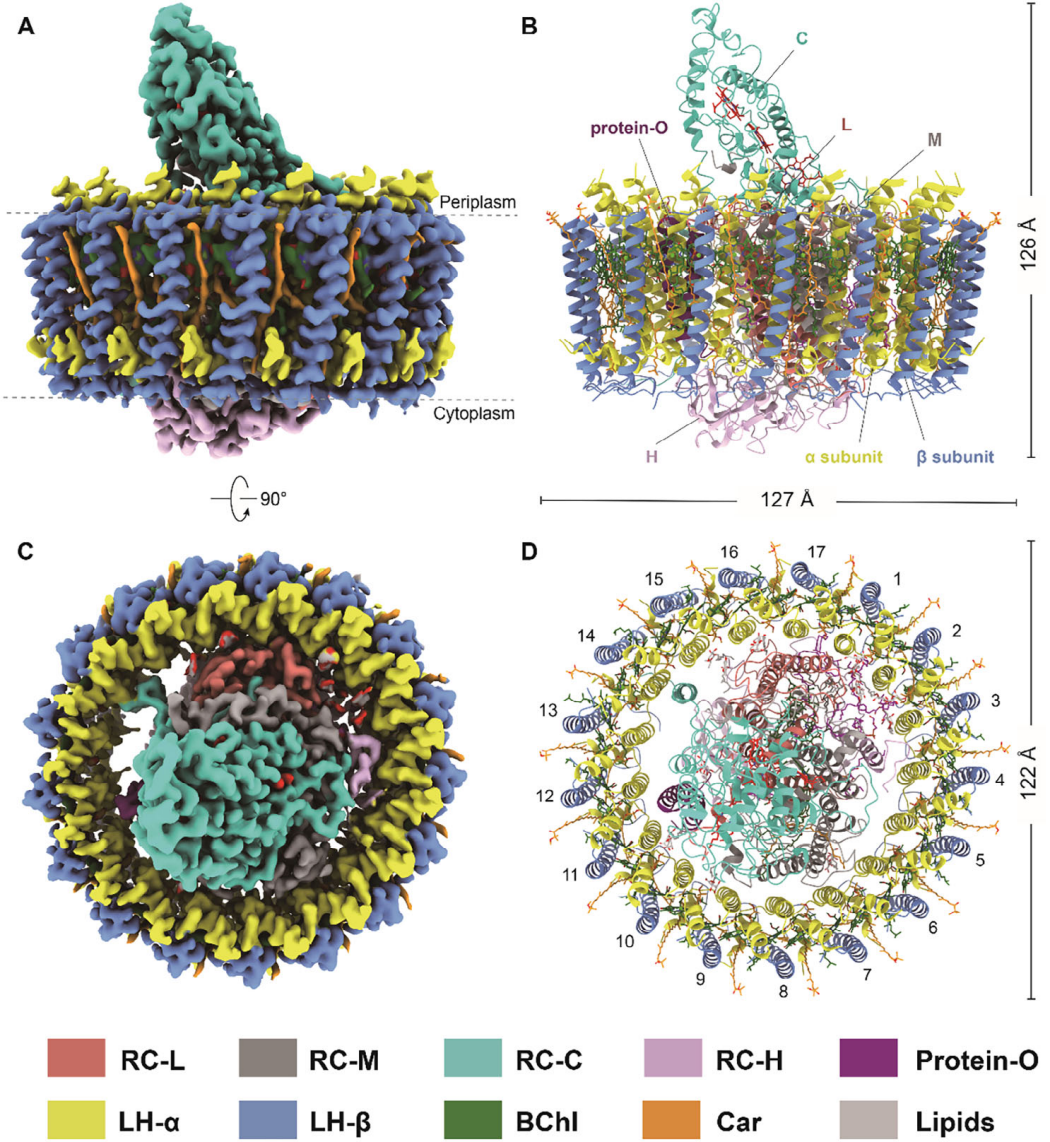
Cryo‐EM structure of *Ds*RC‐LH1. A, C) Electron density map and B, D) atomic structure of *Ds*RC‐LH1 with views from the plane of the membrane (A, B) and the periplasmic side (C, D). The color code is shown below the panels.

The *Ds*RC‐LH1 complex exhibits overall dimensions of ≈127 Å by 122 Å by 126 Å (**Figure** **1**). It consists of a type‐2 RC with four subunits (LMHC) surrounded by a closed LH1 ring composed of 17 αβ heterodimers (Figure 1B,D). Both the L and M subunits consist of five transmembrane helices (TMHs) (Figure 1D, Figure S4, Supporting Information). The C subunit attaches to the surface of the LM heterodimer with its N‐terminal TMH inserted into the membrane, while the heme‐binding domain extends into the periplasmic space (Figure 1). The subunit H associates with the LM heterodimer on the cytoplasmic side, with its N‐terminal TMH extending into the gap between the RC and LH1 (Figure 1B,D). Within the cavity between subunit L and the 11th and 12th α subunits, a strong density with distinct structural features of a “helix‐turn‐helix” motif was observed. This novel subunit, identified as an uncharacterized protein (GenBank accession: ABV93056.1) based on the LC‐MS/MS results (Table S2, Supporting Information), has been designated as protein‐O in the Protein Data Bank (PDB) file (Figure 1B,D).

The architecture of the *Ds*RC‐LH1 is similar to that of the purple bacterial *Blastochloris* (*Blc*.) *viridis* RC‐LH1,^[^ ^15^ ^]^ which also consists of a four‐subunit RC and a closed LH1 ring composed of 17 αβ heterodimers (Figure 1D; Figure S5, Supporting Information). However, the *Blc. viridis* RC‐LH1 binds an additional 16 γ apoproteins, which are tightly packed in the intervals of β subunits outside the LH1 ring, resulting in a compact pigment arrangement (Figure S5, Supporting Information). The sequence identities between the subunits L, M, C, H, α, and β of the *Ds*RC‐LH1 and those of the *Blc. viridis* RC‐LH1 are 65%, 52%, 32%, 39%, 53.66%, and 40.82%, respectively, and the Cα root‐mean‐square deviation (RMSD) between the protein‐O‐deleted *Ds*RC‐LH1 and γ apoprotein‐deleted *Blc. viridis* RC‐LH1 is 2.96 Å (Figure S5, Supporting Information), indicating a high similarity in their structures.

In addition to the protein subunits, we identified 38 BChls *a*, two BPhes *a*, 35 spheroidenones, 14 lipids (12 phosphatidylglycerols and two di‐phosphatidylglycerol), three heme groups, two ubiquinone‐10 molecules, and one iron atom within the *Ds*RC‐LH1 (Figure S4, Supporting Information).^[^ ^16^ ^]^ These ligands, in conjunction with the protein subunits, contribute to a total molecular weight of ≈430 kDa for *Ds*RC‐LH1.

### 2.2 Cytochrome *c* Subunit

**Figure 2:**
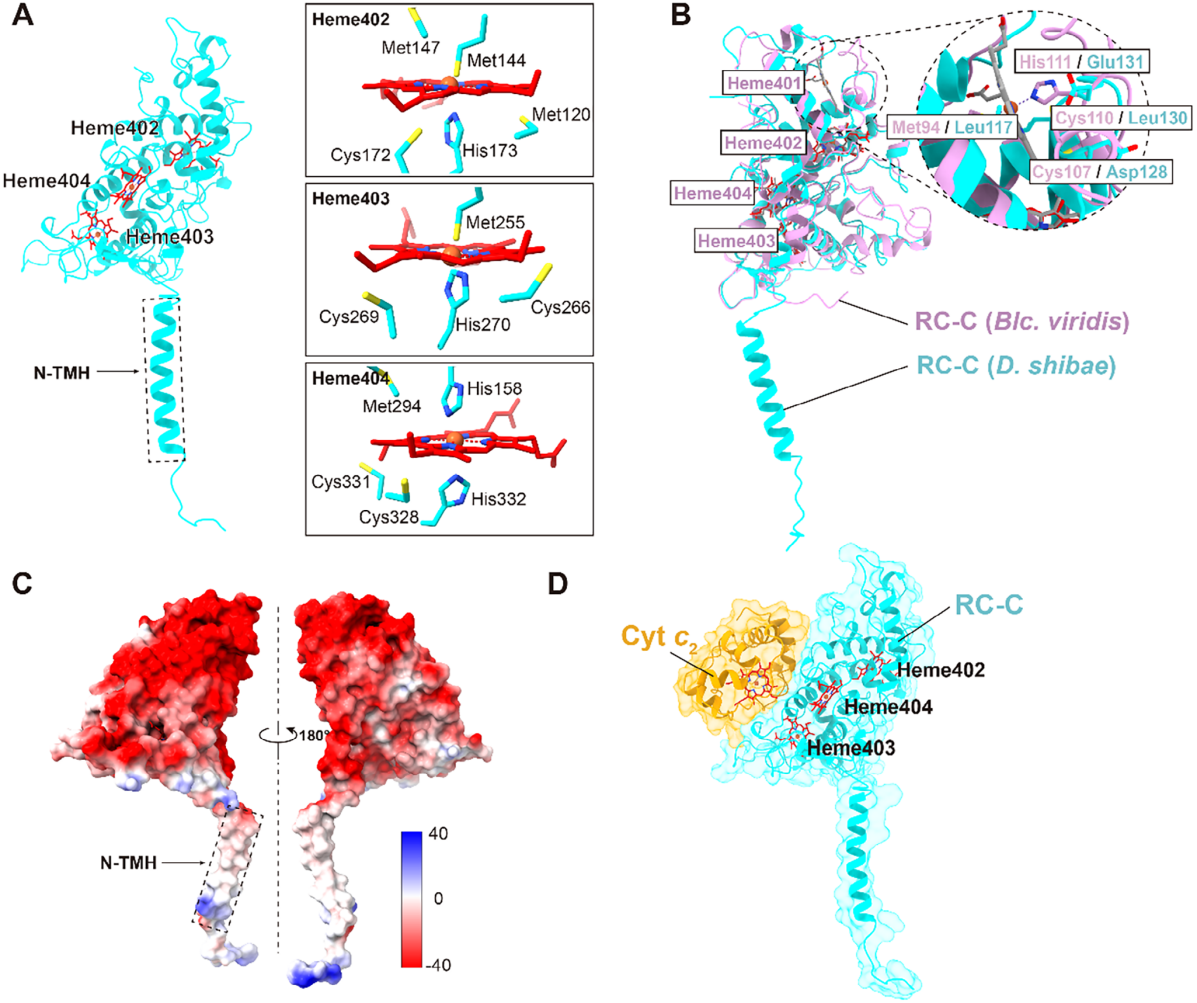
Structure of *Ds*RC‐C. A) *Ds*RC‐C contains an N‐terminal TMH and a C‐terminal heme‐binding domain. The residues that coordinate the heme cofactors are indicated in the panels on the right. B) Superposition of the heme‐binding domains of *Ds*RC‐C (cyan) and *Blc. viridis* RC‐C (pink). The dashed circle indicates the binding site of heme‐401 in *Blc. viridis* RC‐C and a counterpart heme are absent from this region in the *Ds*RC‐C due to their different local residue environments. C) The surface electrostatic potential of *Ds*RC‐C. D) Predicted model of *Ds*Cyt *c*_2_‐*Ds*RC‐C by AlphaFold 3. The Cyt *c*_2_ and *Ds*RC‐C subunits are colored in orange and cyan, respectively.

The cytochrome *c* subunit of the *Ds*RC (*Ds*RC‐C) consists of an N‐terminal TMH (N‐TMH) and a C‐terminal heme‐binding domain, exhibiting an overall structure similar to that of the RC‐C subunits of purple bacteria (**Figures** 1B and **2**A).^[^ ^5^ ^]^ However, the *Ds*RC‐C is distinct in that it contains only three heme groups, specifically numbered 402, 403, and 404, with the equivalent of 401 missing (**Figure** **2**A,B; Figure S6A, Supporting Information). All heme groups within *Ds*RC‐C are hexacoordinated and stabilized by “CXXCH” motifs with the sixth ligand being another histidine or methionine (Figure 2A). Notably, the pocket structure that harbors the first heme (heme 401) in the *Blc. viridis* RC‐C is absent in the *Ds*RC‐C (Figure 2B).^[^ ^15^ ^]^ In particular, the residues Met^94^, Cys^107^, Cys^110^, and His^111^ coordinate heme‐401 in the *Blc. viridis* RC‐C are replaced by Leu^117^, Asp^128^, Leu^130^, and Glu^131^ in the *Ds*RC‐C (Figure 2B; Figure S7, Supporting Information), which cannot support the binding of a heme. The heme‐binding domain of the *Ds*RC‐C has a surface extensively populated with acidic residues, leading to a negative surficial electrostatic potential, which may facilitate the binding of periplasmic electron donors (Figure 2C).

The N‐TMH of *Ds*RC‐C is inserted into the gap between subunit L and the LH ring, establishing robust hydrophobic interactions with the 14th and 15th LH α subunits (Figure S8, Supporting Information). The localization of the N‐TMH of *Ds*RC‐C differs from that observed in the RC‐C subunits of purple bacteria such as *Rhodopila* (*Rpi*.) *globiformis* and *Roseiflexus* (*Rfx*.) *castenholzii* ^[^ ^17^ ^]^ (Figure S9, Supporting Information). The N‐TMH of *Rfx. castenholzii* RC‐C is inserted into the LH1 ring and occupies the corresponding position of one LH α subunit (Figure S9, Supporting Information). In contrast, the N‐TMH of *Rpi. globiformis* RC‐C is inserted into the large cavity between the RC and LH ring and interacts extensively with residues from the RC‐M and H, as well as a nearby LH1 α‐polypeptide on the cytoplasmic side (Figure S9, Supporting Information). These distinct arrangements of the N‐TMHs of RC‐C subunits may reflect functional differences.

To explore the potential electron transfer mechanism from soluble Cyt *c*_2_ to the *Ds*RC, we constructed a chimeric *Ds*Cyt *c*_2_‐*Ds*RC‐C model using AlphaFold 3^[^ ^18^ ^]^ (Figure 2D, Figures S10 and S11A,B, Supporting Information). In this model, Cyt *c*_2_ is attached to two helices (ranging from Trp^147^ to Gly^157^ and Thr^282^ to Asn^300^) and a loop (ranging from Ala^274^ to His^281^) of *Ds*RC‐C (Figure 2D; Figure S11A,B, Supporting Information). The Fe‐Fe distance between the heme group of Cyt *c*_2_ and heme 403 of *Ds*RC‐C is 18.4 Å, which is shorter than the distances (32 and 19.4 Å) between the Fe atoms of Cyt *c*_2_ and hemes 402 and 404, and similar to the Fe‐Mg distances (20.4 and 20.8 Å) between heme 403 and the SP in the *Ds*RC (Figure S11C, Supporting Information). This indicates that electron transfer from Cyt *c*_2_ to the SP is through heme 403 within Cyt *c*_2_‐*Ds*RC system.

### 2.3 The Newly Identified Protein‐O

**Figure 3:**
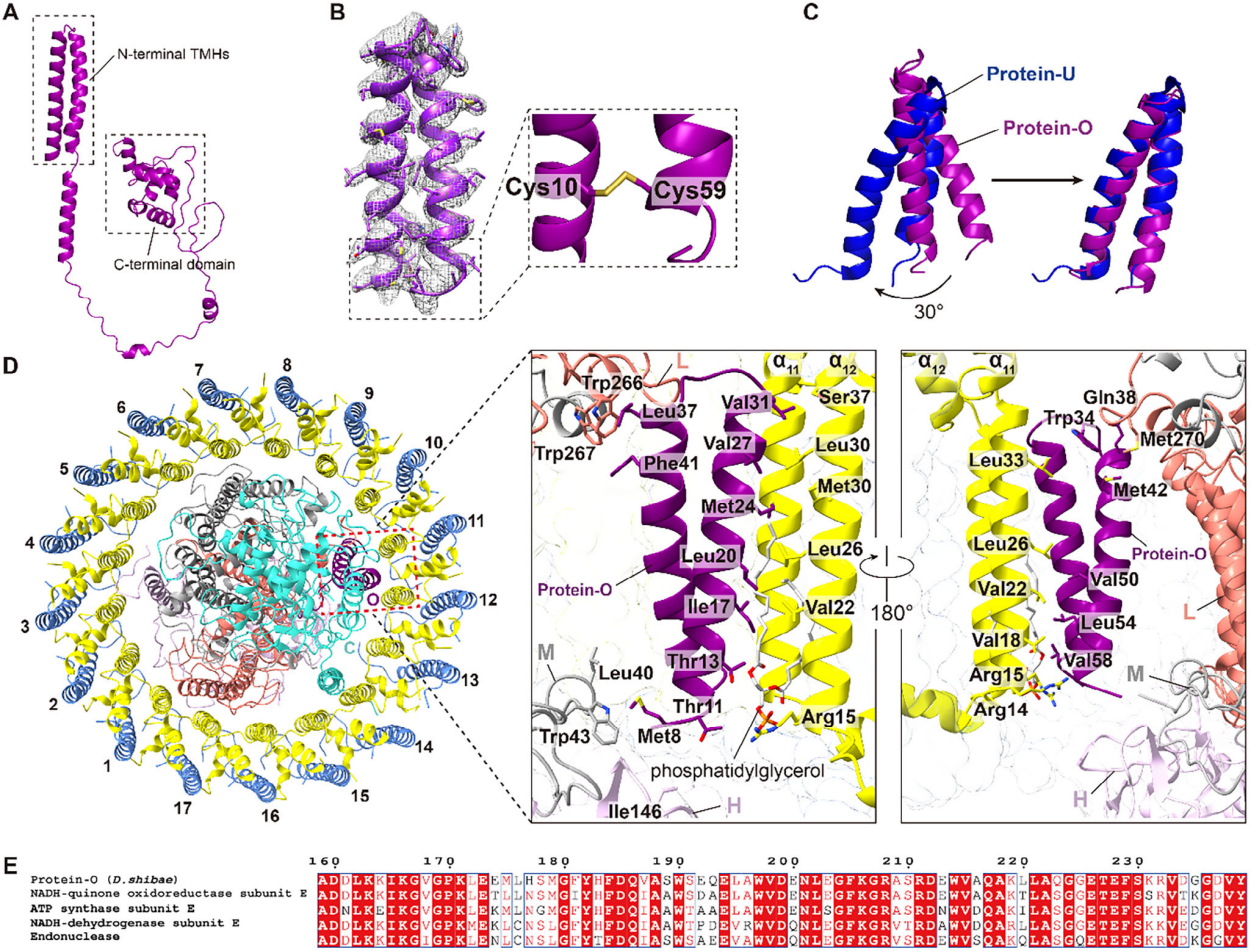
Structural analysis of protein‐O. A) Overall structure of the full‐length protein‐O predicted by AlphaFold3. B) The density of the N‐terminal TMHs of protein‐O with the disulfide bond enlarged. C) Structural alignment of the N‐terminal TMHs of protein‐O and protein‐U. The two structures can be overlapped by rotating 30 degrees. D) Interactions between protein‐O and its surrounding subunits colored as Figure 1. Residues at the interfaces are indicated. E) Sequence alignment of the C‐terminal domains of protein‐O, NADH‐quinone oxidoreductase subunit E, ATP synthase subunit E, NADH‐dehydrogenase subunit E, and endonuclease from *D. shibae*, *Roseovarius*, *Rhodobacteraceae*, *Roseivivax* and *Thalassococcus profundi*.

The full‐length sequence of protein‐O comprises 239 residues, with a molecular weight of 25177 Da (Figure S12, Supporting Information). The predicted structure of protein‐O by AlphaFold 3 consists of an N‐terminal transmembrane domain (NTD) and a C‐terminal short‐helix‐rich domain connected by a long flexible loop^[^ ^18^ ^]^ (**Figure** **3**A). Only the residues from Met^8^ to Cys^59^ of the NTD could be traced in the current cryo‐EM map and are characterized by a “helix‐turn‐helix” motif, however, perhaps because the remaining segments are flexible and are invisible in the map or may have been lost during sample preparation (Figure 3B, Figure S4, Supporting Information). Notably, a disulfide bond is observed between residues Cys^10^ and Cys^59^ within the TMHs on the cytoplasmic side, resulting in an O‐shaped conformation of the NTD (Figure 3B). The architecture of the NTD of protein‐O resembles that of protein‐U (or protein‐Y) in the *Rba. sphaeroides* RC‐LH1 with their Cα RMSD less than 1.8 Å, and they are located at similar positions in the space between the RC and LH1^[^ ^19^ ^]^ (Figure 3C; Figure S13A, Supporting Information). However, their primary sequences are quite distinct, and no genes encoding homologs of protein‐U were identified in the genome of *D. shibae* (Figure S13B, Supporting Information).

Extensive interactions are identified between the protein‐O NTD, RC, and LH1 in the *Ds*RC‐LH1 (Figure 3D). Specifically, the NTD interacts with the TMHs of the 11th and 12th LH α subunits through hydrophobic interactions between nonpolar residues at the interfaces (Figure 3D). A blob of density with the characteristic of a phosphatidylglycerol molecule is observed between the NTD and LH1, providing additional interactions (Figure 3D; Figure S5, Supporting Information). Furthermore, the cytoplasmic end of the NTD interacts with two short loops (Leu^40^ to Trp^43^ and Ala^144^ to Ile^146^) from subunits L and H, respectively. The periplasmic end of the NTD interacts with a segment (Trp^266^ to Met^270^) of subunit L (Figure 3D).

The function of protein O remains unknown. Given that the NTD of protein‐O occupies a similar position as protein‐U (Figure 3C; Figure S13, Supporting Information), we speculate that it plays a comparable role in the assembly of the *Ds*RC‐LH1.^[^ ^19^ ^]^ Bioinformatic analysis reveals that the C‐terminal domain (from Ala^159^ to Tyr^239^) of protein‐O exhibits high degrees of sequence identities with the C‐termini of NADH‐quinone oxidoreductase subunit E, ATP synthase subunit E, NADH‐dehydrogenase subunit E and endonuclease of *D. shibae*, *Roseovarius*, *Rhodobacteraceae*, *Roseivivax* and *Thalassococcus profundi*. (Figure 3E; Table S3, Supporting Information). Unfortunately, the physiological functions of these domains are obscure. Interestingly, within the genome of *D. shibae*, the coding sequence of protein‐O is located between the genes encoding NADH‐quinone oxidoreductase subunits E and F but is distant from the coding sequences of the PGC (Figure S1, Supporting Information). This indicates that transcription of the gene encoding protein‐O may be regulated independently of its flanking genes. It remains unclear whether the coding gene of protein‐O was originally possessed by the bacteria itself or was acquired through horizontal gene transfer or gene recruitment.^[^ ^11^, ^14^, ^20^ ^]^

### 2.4 Stabilizing Effects of Mg^2+^ and Ca^2+^ on the *Ds*RC‐LH1 Complex

**Figure 4:**
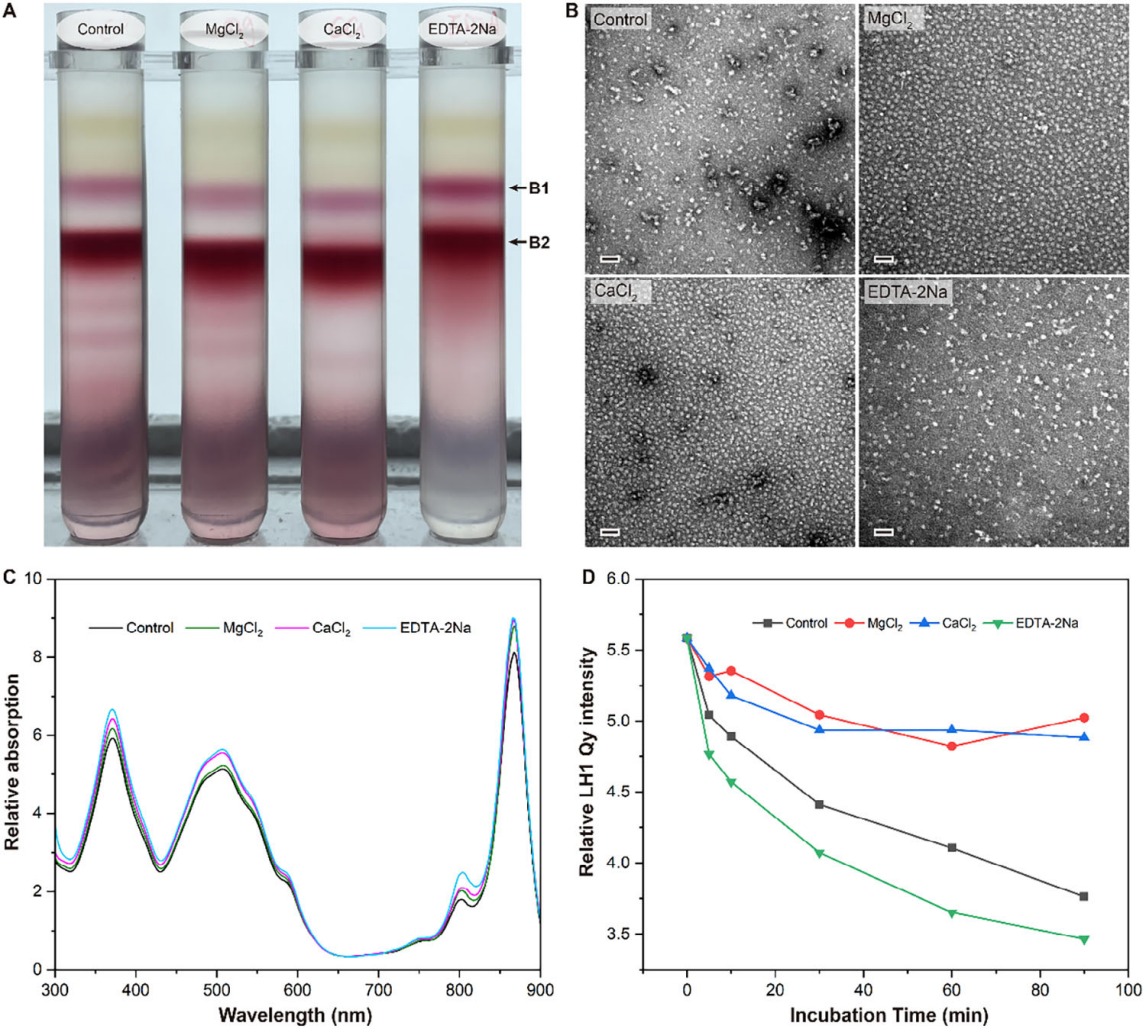
Stabilizing effects of Mg^2+^ and Ca^2+^ on the purified *Ds*RC‐LH1 complex. A) Sucrose density gradient centrifugation of DDM‐solubilized *Ds*RC‐LH1 samples in salt‐free (control), 10 mM MgCl_2_, 10 mM CaCl_2_, and 10 mM EDTA‐2Na solutions, respectively. The deep reddish‐purple bands (B2) were enriched in the *Ds*RC‐LH1 complex and collected for further analysis. B) Negative staining electron microscopy of the *Ds*RC‐LH1 samples purified under different conditions as shown in (A). The scalebar within each panel corresponds to 50 nm. C) Room‐temperature absorption spectra of the purified *Ds*RC‐LH1 samples. The spectra were normalized at 651 nm. D) Changes of the relative LH1‐Q_y_ absorption intensity of the purified *Ds*RC‐LH1 samples as functions of time. The samples were incubated at 50 °C.

The purified *Ds*RC‐LH1 complex showed high stability in Mg^2+^‐ or Ca^2+^‐containing solutions (**Figure** **4**). When equal amounts of cell membranes were dissolved in solutions lacking salt, or containing Mg^2+^, Ca^2+^, or ethylenediaminetetraacetic acid disodium salt (EDTA‐2Na), and separated by sucrose density gradient centrifugation (SDGC), the *Ds*RC‐LH1‐rich components formed deep reddish‐purple bands at similar locations in the gradient tubes (Figure 4A), and their absorption spectra were similar (Figure 4C). However, the negative staining analysis showed that the stabilities of the *Ds*RC‐LH1 samples purified with different conditions were significantly different (Figure 4B). In particular, the *Ds*RC‐LH1 particles exhibited stable forms in Mg^2+^ and Ca^2+^ solutions, whereas most of the *Ds*RC‐LH1 particles purified in salt‐free or EDTA‐2Na solutions were severely disrupted (Figure 4B). When incubated at 50 °C, the *Ds*RC‐LH1 samples in Mg^2+^ and Ca^2+^ solutions showed higher thermal stability and the relative intensity of LH1 Q_y_ absorption at 867 nm decreased slightly within 90 min (Figure 4D; Figure S14, Supporting Information). However, the *Ds*RC‐LH1 samples in salt‐free or EDTA‐2Na solutions exhibited a significant decrease in the relative intensity of LH1 Q_y_ absorption (Figure 4D; Figure S14, Supporting Information). There is no remarkable difference between the overall structures of the *Ds*RC‐LH1 complexes purified in salt‐free, Mg^2+^, and EDTA‐2Na solutions (Figures S3 and S15,16, Supporting Information). Interestingly, we observed two additional densities (site1 and site2) which are located at the interfaces between RC‐M, RC‐C, and the 8–9th LH α subunits only in the *Ds*RC‐LH1 sample purified using the Mg^2+^ solution (Figure S17, Supporting Information). However, since the distances between these two densities and their surrounding residues exceed 3.5 Å, water molecules could potentially form intermolecular hydrogen bonds, facilitating their arrangement in a cohesive manner (Figure S17, Supporting Information).

### 2.5 Arrangement of Pigments and Lipids

The BChl arrangement in the *Ds*RC‐LH1 is very similar to that observed in the *Blc. viridis* RC‐LH1.^[^ ^15^ ^]^ When viewed parallel to the membrane plane, the LH BChls in the *Ds*RC‐LH1 are densely packed near the periplasmic side, with the porphyrin rings nearly perpendicular to the membrane plane. The central magnesium atoms are positioned at approximately the same level, with slight fluctuations in either upward or downward positions (Figure S6A, Supporting Information). Notably, when superimposing the central L subunit of *Ds*RC‐LH1, *Rba. sphaeroides* RC‐LH1 and *Blc. viridis* RC‐LH1, the *Ds*LH1 ring aligns nicely with the *Rs*LH1 ring.^[^ ^15^, ^19^ ^]^ Their BChl‐Mg planes exhibit a tilt of ≈5 degrees compared to that of the *Blc. viridis* LH1 ring (Figure S6A, Supporting Information). This slight tilt in the BChl‐Mg plane may be attributed to specific areas that facilitate the diffusion of quinones and quinols across the *Ds*LH ring or the asymmetrical dimerization of the *Rba. sphaeroides* RC‐LH1s.^[^ ^19^, ^21^ ^]^

When viewed perpendicular to the membrane plane, the LH BChls are distributed in an ellipse with a long axis of 97 Å and a short axis of 93 Å (Figure S6B, Supporting Information). The Mg‐Mg distances between the *Ds*RC BChls and the closest LH BChls show great variation, ranging from 33.8 to 50.0 Å, compared to the narrower range observed in the *Blc. viridis* RC‐LH1 (35.2 to 46.2 Å)^[^ ^15^ ^]^ (Figure S6B, Supporting Information). Remarkably, the shortest distance from LH BChls to the SP BChls is 38.9 Å, measuring from the 5th heterodimer BChls (Figure S6B, Supporting Information). However, this asymmetric BChl arrangement around *Ds*RC may have no remarkable effects on the uphill energy transfer from LH1 to the RC, which is mainly optimized by the thermal activation mechanism.^[^ ^22^ ^]^ Of course, factors other than proximity contribute to the pathway of excitation energy transfer (EET). The EET from LH1 to the RC can likewise be facilitated via the “passage‐forming BChls” that possess preferential geometric orientations toward the SP along the LH1 ring, as previously reported.^[^ ^22a^ ^]^ The Mg‐Mg distances between the BChls of two adjacent LH αβ heterodimers range from 7.6 to 8.6 Å, which are slightly shorter than the Mg‐Mg distances (9.0 to 9.7 Å) between the BChls within each LH αβ heterodimer (Figure S18, Supporting Information). This suggests an efficient EET and equalization among the LH BChls.

The electron transfer chain (ETC) in the *Ds*RC comprises two pairs of BChls *a*, two BPhes *a*, and two ubiquinone‐10 molecules, which are symmetrically arranged in two branches (designated as branch A and branch B), similar to the configuration observed in purple bacterial RCs.^[^ ^5^ ^]^ The primary electron donor is a special pair of BChls *a* coordinated by histidine residues (His^174^ and His^203^) from subunits L and M, respectively (Figure S19, Supporting Information). The two quinones, Q_A_ and Q_B_, located near the BPhes *a*, serve as the primary and secondary electron acceptors, respectively, with their polar heads embedded within the LM heterodimer. The head of Q_B_ is in a more hydrophilic environment than that of Q_A_ (Figure S19, Supporting Information). Multiple sequence alignment analysis revealed that the surrounding residues at the quinone‐binding sites are highly conserved among RCs from different phototrophs (Table S4; Figure S19, Supporting Information). An additional conserved quinone (Q_P_) had been found in several RCs and its location was regarded as an indicator of the possible diffusion path for quinones.^[^ ^5^, ^23^ ^]^ Unfortunately, we did not identify additional electron density corresponding to a third quinone molecule within the *Ds*RC‐LH1 complex. This absence could be attributed to the relatively weak binding of the putative third quinone, which may have led to its dissociation and subsequent loss during specimen preparation.

Spheroidenone, a ketolated derivative of spheroidene, is the exclusive carotenoid identified in *D. shibae* cells and exhibits characteristic absorption peaks centered at 506 nm (Figure S2D, Supporting Information).^[^ ^13^, ^24^ ^]^ The arrangement of spheroidenones in the *Ds*RC‐LH1 is highly similar to that of spheroidenes in the *Rba. sphaeroides* RC‐LH1 (Figure S20A, Supporting Information).^[^ ^19c^ ^]^ Along the *Ds*LH ring, each LH αβ heterodimer accommodates two spheroidenones, with their polar heads exposed to the solvent and alkyl hydrocarbon tails embedded in the membrane (Figure S20B, Supporting Information). The polyene skeletons of spheroidenones interact closely with the BChl porphyrin ring (Figure S20B, Supporting Information), and this densely packed arrangement of carotenoids within the *Ds*RC enhances light absorption and stabilizes the RC‐LH1 complex by quenching reactive oxygen species generated by triplet‐state BChls in aerobic environments.^[^ ^25^ ^]^

The majority of lipids that purified with the *Ds*RC‐LH1 are situated at the space between the N‐TMH of subunit H, the first TMH of subunit L, and the 1st‐3rd LH αβ heterodimer (Figure 1D). These lipids presumably play critical roles in stabilizing the *Ds*RC‐LH1 complex.

### 2.6 Quinone/Quinol Diffusion within *Ds*RC‐LH1

**Figure 5:**
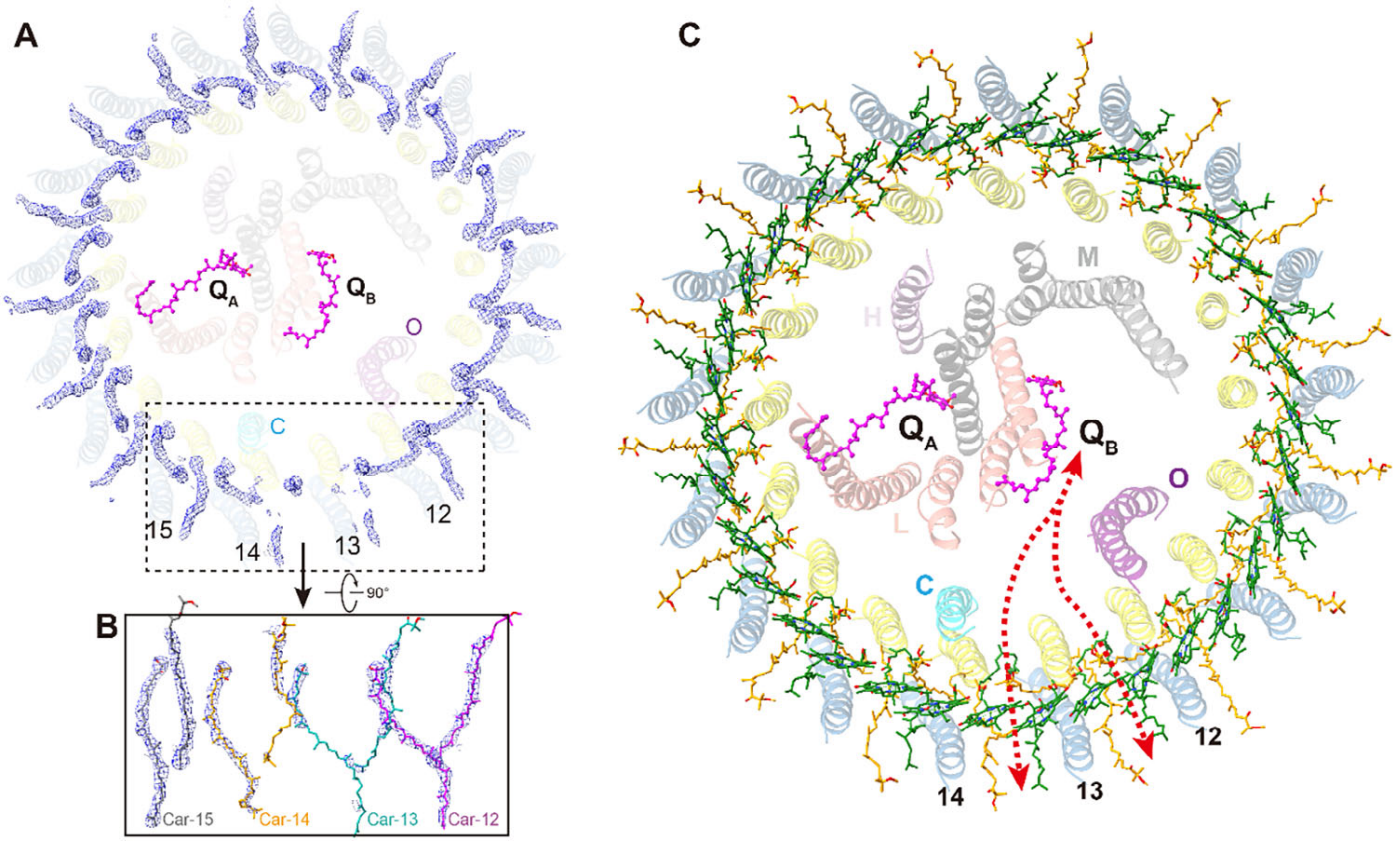
Proposed quinone/quinol diffusion pathways in the *Ds*RC‐LH1 complex. A) Periplasmic view of the *Ds*RC‐LH1 complex with semi‐transparent cartoon representation, showing the densities (blue mesh) for spheroidenones within the LH1 ring. Q_A_ and Q_B_ are depicted as magenta ball‐and‐stick. B) Membrane plane visualization of four spheroidenone pairs spanning LH αβ heterodimers 12–15. The densities for the spheroidenones associated with the 12th (purple), 13th (cyan), 14th (orange), and 15th (gray) LH heterodimers exhibit different profiles. C) Proposed quinone/quinol diffusion pathways. The gaps between the 12th and 13th, 13th, and 14th LH heterodimers are proposed to be the quinone/quinol exchange sites, and the putative diffusion pathways are shown as red dashed arrows.

The detailed mechanism of quinone/quinol diffusion within RC‐LH1 complexes has been a matter of intense discussion.^[^ ^5^ ^]^ In RC‐LH1s with open LH rings, quinones/quinols are thought to shuttle primarily through the relatively large gap in the ring of the LH αβ heterodimers.^[^ ^5^ ^]^ In contrast, in RC‐LH1s with closed LH rings forming a palisade around the RC, quinones/quinols are proposed to diffuse through relatively small channels between two adjacent LH αβ heterodimers near the cytoplasmic side.^[^ ^5^, ^26^ ^]^ The *Ds*RC‐LH1 exhibits striking structural similarity to the *ΔpufX* RC‐LH1 monomer isolated from *Rba. sphaeroides pufX*‐deletion mutants.^[^ ^19b^ ^]^ In this modified architecture, the RC is surrounded by a closed LH1 ring composed of 17 αβ‐polypeptide subunits.^[^ ^19b^ ^]^ Notably, an accessory carotenoid molecule bound between adjacent LHαβ subunits generates steric constraints that effectively block the quinone/quinol translocation channel across the LH1 barrier, thereby explaining the observed photosynthetic deficiency in this mutant strain.^[^ ^19b^ ^]^ Intriguingly, while the carotenoid arrangement pattern in *Ds*LH1 resembles that of the *ΔpufX* RC‐LH1 monomer, our density map analysis demonstrates significantly attenuated electron densities for two carotenoid pairs positioned between the 12th and 14th LH αβ heterodimers, in contrast to the well‐defined densities observed for other carotenoids along the LH1 ring (**Figure** **5**A,B). This localized structural disorder cannot be attributed to interactions between the N‐TMH of the *Ds*RC‐C and the 14th /15th LH1 α subunits, as the carotenoid densities between the 14th and 15th LH αβ heterodimers remain comparable to those of other regions. These observations suggest a potential mechanism whereby quinones may traverse the *Ds*LH1 ring through restricted intersubunit gaps formed between the 12th and 13th, 13th and 14th LH αβ heterodimers (Figure 5C). In this case, the NTD of protein‐O interacts with the L subunit to form an internal pathway for quinone molecules to reach the Q_B_ site (Figure 5C).

## 3 Discussion

As a basic functional unit of photosynthesis, RC‐LH1s have been extensively studied, resulting in the determination of numerous RC‐LH1 structures.^[^ ^5^ ^]^ However, the *Ds*RC‐LH1 structure reported here exhibits distinct characteristics, such as a three‐heme Cyt *c* subunit and a newly identified protein‐O. These properties distinguish the *Ds*RC‐LH1 from previously reported RC‐LH1s. The structure of *Ds*RC‐LH1 may represent an evolutionary stage between RCs that contain a tetraheme Cyt subunit and those that lack a multi‐heme bound Cyt. This is consistent with the observation that purple bacteria lacking the Cyt subunit exhibit significant aerobic growth, which is rarely observed in purple bacteria containing a Cyt subunit‐binding RC.^[^ ^8^ ^]^

**Figure 6:**
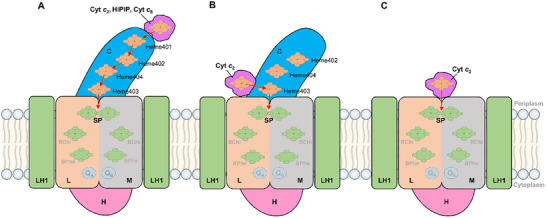
Comparison of electron transfer chains in different RC‐LH1s. A) For RC‐LH1s bound with a tetra‐heme RC‐C subunit, the soluble electron donor (Cyt *c*_2_, HiPIP, Cyt *c*_8_) binds near the distal heme (heme‐1), and electrons are transferred along the hemes to the SP. B) For RC‐LH1s that bound with a tri‐heme RC‐C subunit, as exhibited by the predicted *Ds*Cyt *c*_2_‐RC‐LH1 model, the periplasmic electron donor (Cyt *c_2_*) possibly binds to the RC‐C subunit near the membrane plane, and the electrons are transferred directly to heme‐3, and thence to the SP. C) For RC‐LH1s lacking an RC‐C subunit, the periplasmic electron donor (Cyt *c_2_*) binds to the periplasmic surface of the RC‐LM dimer, and electrons are transferred directly to the SP.

The distinct arrangement of heme groups in the *Ds*RC‐C indicates a different electron transfer pathway within the *Ds*RC‐LH1 compared to the tetraheme Cyt *c*‐binding RCs like those found in *Thermochromatium* (*Tch*.) *tepidum* and *Blc. viridis* (**Figure** **6**).^[^ ^27^ ^]^ In these tetraheme Cyt *c*‐binding RCs, soluble electron donors such as Cyt *c*_2_ and the high‐potential iron‐sulfur protein (HiPIP) attach to the solvent‐accessible edge of heme 401, which is located farthest from the SP, proceeding with uphill electron transfer from Cyt *c*_2_ to heme 403 (the heme closest to the SP)^[^ ^28^ ^]^ (Figure 6). However, due to the absence of the corresponding heme 401 in the *Ds*RC‐LH1, electron transfer from Cyt *c*_2_ to the SP is predicted to be via heme 403 according to the model for the *Ds*RC‐LH1 (Figure 6). Notably, the Fe‐Mg distances (20.4 Å and 20.8 Å, Figure S11C, Supporting Information) between heme 403 and the SP in the *Ds*RC‐LH1 closely match the distances (21.2 Å and 21.3 Å) in the homologous *Rba. sphaeroides* Cyt *c*_2_‐RC‐LH1 crystal structure (PDB: 1L9J). Similar distances could be found in the *Blc. viridis* (21 Å and 21.1 Å, PDB: 6E5T) and *Tch. tepidum* (20.3 and 20.7 Å, PDB: 5Y5S) RC‐LH1 structures, which also have a multi‐heme cytochrome bound to the RC. This implies that the farthest heme (heme 402) in the *Ds*RC‐LH1 may not be directly functional in mediating electron transfer. In addition, heme 402 is almost embedded in the *Ds*RC‐C, hindering the acquisition of electrons from Cyt *c*_2_. Nevertheless, it should be noted that efficient electron transfer requires rapid dissociation of Cyt *c*_2_ from the bound Cyt subunit, and their binding must be influenced by both their redox potentials and side‐chain interactions. We tried to isolate the Cyt *c*_2_‐*Ds*RC‐LH1 complex in vitro but were unsuccessful, indicating that the binding of Cyt *c*_2_ to *Ds*RC‐LH1 is weak. The predicted Cyt *c*_2_‐*Ds*RC‐LH1 model by AlphaFold 3 favors electron transfer from Cyt *c*_2_ to the SP via heme 403, however, the confidence metrics (pTM = 0.71, ipTM = 0.69) of the model imply that other interactions are possible.^[^ ^29^ ^]^ Currently, the potentials associated with each heme group within *Ds*RC‐C are unknown, so a complete elucidation of the electron transfer mechanisms awaits further kinetic examination combined with mutational analyses in the context of the Cyt *c*_2_‐*Ds*RC‐LH1 structure reported here.

The highly comparable overall architectures of the RC‐LH1s in *D. shibae* and *Blc. viridis* strongly support the hypotheses that AAPB evolved directly from a purple nonsulfur bacterial (PNSB)‐like ancestor through vertical evolution, or from a heterotrophic bacterial ancestor via horizontal evolution by acquiring the PGC from PNSB.^[^ ^12^ ^]^ However, unlike most PNSB that preferentially perform anoxygenic photosynthesis under anaerobic conditions,^[^ ^30^ ^]^ *D. shibae* is capable of performing anoxygenic photosynthesis only in the presence of oxygen.^[^ ^13^ ^]^ Similar to most AAPB, *D. shibae* cells synthesize a significant amount of carotenoids (spheroidenones) that neutralize triplet BChl *a* and singlet oxygen generated during BChl *a* excitation. The N‐TMH of protein‐O within *Ds*RC‐LH1 has a similar overall structure to that of protein‐U (or protein‐Y) in the *Rba. sphaeroides* RC‐LH1,^[^ ^19^ ^]^ However, they exhibit different postures in the space between the RC and LH1. The disulfide bond in the N‐TMH of protein‐O is unique in *Ds*RC, and had not been found in the structures of other photosynthetic bacterial RCs. Considering the flexible soluble C‐terminal domain of protein‐O, we propose that this disulfide bond functions to stabilize the N‐TMH of protein‐O.

It has been widely accepted that salt has a significant impact on the stability of proteins by modifying the ionic strength of the solution. In several purple bacteria, Ca^2+^ ions have been found to bind at the interfaces of adjacent LH αβ heterodimers,^[^ ^31^ ^]^ contributing to the thermo‐stability of LH1 and a redshift of the absorption peak of RC‐LH1s.^[^ ^26^ ^]^ Within the *Tch. tepidum* RC‐LH1, each Ca^2+^ is coordinated by four residues (α_n_‐Trp^46^, α_n_‐Asp^49^, α_n_‐Ile^51^, β_n+1_‐Trp^45^) and two water molecules. However, in the *Ds*RC‐LH1 the residues α_n_‐Asp^49^ and α_n_‐Ile^51^ are replaced by α‐Leu^46^ and α‐Ala^47^, resulting in the inability of Ca^2+^ to be stably bound at this position (Figure S21, Supporting Information). This is consistent with the observation that the *Ds*RC‐LH1 samples purified using Ca^2+^‐ or Mg^2+^‐containing solutions showed no significant changes in the absorption spectra compared to *Ds*RC‐LH1 samples purified using salt‐free or EDTA‐2Na solutions (Figure 4). Since we found only two possible binding sites of bivalent ions in the *Ds*RC‐LH1 complex, the stabilizing effects of bivalent ions on the complex may be mainly through non‐specific binding to the surface of the complex or the residues distributed in the gap between the reaction center and the LH1, which may also represent an adaptation to the marine environments where *D. shibae* has been found to exist.^[^ ^13^ ^]^

While the quinone shuttling between the Q_B_ site of the RC and the Cyt *bc*_1_ complex is critical for sustaining the photosynthetic electron transport chain, purple photosynthetic bacteria inhabiting distinct ecological niches have evolved divergent structural and kinetic adaptations to optimize this process.^[^ ^5^ ^]^ In non‐enclosed RC‐LH1 complexes, quinones achieve efficient shuttling through open gaps in the LH1 ring. In contrast, most closed RC‐LH1 systems, which exhibit a 1:1 stoichiometric binding of BChls and carotenoids within the LH ring, rely on a “breathing motion” of the LH1 scaffold or transiently formed small gaps to enable quinone diffusion.^[^ ^5^ ^]^ Notably, *Gem. phototrophica* possesses a double‐layered closed LH architecture, which presents a significant steric hindrance to quinone transport.^[^ ^5^, ^23^ ^]^ However, the larger polypeptide spacing in its outer LHh ring confers enhanced structural flexibility, allowing quinones to diffuse via structural dynamics or localized gaps.^[^ ^5^, ^23^ ^]^ Similarly, the RC‐LH1 complexes of *Blc. viridis* and *Rpi. globiformis*, despite binding gamma‐like extrinsic polypeptides peripherally, retain unsealed gaps that permit quinone diffusion.^[^ ^15^, ^17^ ^]^ In the case of *Ds*RC‐LH1, the binding of more LH1 carotenoids would offer enhanced photoprotection and the capacity to absorb and use more solar energy, but at the same time bear the risk of quinone diffusion being affected. The precise mechanism of quinone shuttling in *Ds*RC‐LH1 remains uncertain. Based on structural evidence, we hypothesize that quinones in *Ds*RC‐LH1 may traverse the closed LH1 ring via “breathing motion” or the gaps between the 12th and 13th, 13th and 14th LH αβ heterodimers, a strategy likely refined through long‐term evolutionary adaptation. Furthermore, we propose that the N‐TMH of *Ds*RC‐C serves dual roles: 1) maintaining proper interactions between the RC and LH1, and 2) acting in conjunction with protein‐O and the RC‐L subunit to establish a stable internal conduit, thereby facilitating directional quinone shuttling.

## 4 Experimental Section

### Purification of RC‐LH1 Complex

The *Ds*RC‐LH1 complex was isolated and purified as previously described with some modifications.^[^ ^32^ ^]^ Wild‐type *D. shibae* DFL12^T^ DSM‐16493 [Deutsche Sammlung von Mikroorganismen und Zellkulturen (DSMZ), Germany] cells were grown in 1‐liter glass flasks bubbled with air for three days under white light illumination at 2400 lx and 28 °C. To avoid light‐induced damage to the RC‐LH1 complex, all operations were performed under subdued illuminations. Cells were harvested by centrifugation at 2000 × g for 20 min at 4 °C, and the pellet was resuspended to a concentration of 0.2 g mL^−1^ in a Tris buffer (20 mM Tris‐HCl, pH 8.0) supplemented with DNase I and 10 mM MgCl_2_. Subsequently, the cells were disrupted 6 times at 1600 bars with a cell disruptor (JNBIO) equipped with a cooling system. The resulting cell lysate was centrifuged at 10000 × g for 10 min to eliminate unbroken cells, and the supernatant was collected and centrifuged again at 120000 × g for 40 min. The sediment was resuspended and adjusted to an absorption of 8 at the Q_y_ band (867 nm). The resuspended membranes were solubilized with n‐dodecyl‐β‐D‐maltopyranoside (β‐DDM) at a final concentration of 1% (w/v) for 10 min within a refrigerator at 4 °C. The unsolubilized membrane fragments were removed by centrifugation at 12000 × g for 10 min. The supernatant containing the *Ds*RC‐LH1 complex was loaded onto a continuous sucrose density gradient of 10%–40% (w/v), and centrifuged at 200000×g for 14 h at 4 °C using a SW41Ti rotor with a Beckman Coulter Optima XPN‐100 centrifuge. The buffer contained 0.02% (w/v) β‐DDM and 10 mM MgCl_2_ in 20 mM Tris‐HCl (pH 7.8). Two reddish‐purple bands were obtained from the sucrose density gradient centrifugation (SDGC) (Figure S2, Supporting Information). The upper band (B1) was a mixture of different proteins and featured a maximum absorption at 805 nm (Figure S2, Supporting Information). The lower band (B2) was enriched in *Ds*RC‐LH1 complex and was collected for further purification through an anion exchange column using Q‐Sepharose High Performance media (GE Healthcare). The column was pre‐equilibrated with a low‐salt buffer (20 mM Tris‐HCl, pH 7.8, 10 mM MgCl_2_, 0.02% β‐DDM) before elution with a linear salt gradient (0–1 M NaCl) in the same buffer. The *Ds*RC‐LH1 complex was rich in the peak eluted by 500 mM NaCl. The eluted sample was concentrated to a small volume by centrifugal ultrafiltration using Amicon Ultra filters (100 kDa MWCO), and subjected to a gel filtration column (Superose 6 Increase 10/300, GE). The eluted *Ds*RC‐LH1 complex with a sharp peak was collected and concentrated for subsequent biochemical analysis and cryo‐EM experiments. To study the effects of Mg^2+^ and Ca^2+^ ions on the stability of *Ds*RC‐LH1 complex, we prepared three new batches of *Ds*RC‐LH1 samples with the same protocol described above but used three different solutions: the first group (control, salt‐free) utilized the same solution as described above (Mg^2+^ solution) but lacking Mg^2+^; the second group (Ca^2+^) employed a buffer with 10 mM CaCl_2_ replacing the MgCl_2_; and the third group (EDTA‐2Na) used a solution containing 10 mM EDTA‐2Na. Each batch of *Ds*RC‐LH1 samples was used for subsequent biochemical and EM experiments.

### Biochemical Characterization of RC‐LH1 Complex

The protein composition of purified *Ds*RC‐LH1 complex was analyzed using tricine‐sodium dodecyl sulfate‐polyacrylamide gel electrophoresis (Tricine‐SDS‐PAGE) as follows.^[^ ^33^ ^]^ 5 µL *Ds*RC‐LH1 sample with an absorbance of six for the LH1 Q_y_ band was treated with an equal volume of LDS sample buffer (GenScript, Cat. No. M00676‐250) at 70 °C for 10 min. The denatured sample was centrifugated at 10000 × g for 5 min and then loaded onto a precast mini gel containing 16.5% (w/v) polyacrylamide (Beyotime, Cat. No. P0532S). Electrophoresis was carried out at a constant voltage of 150 V and at room temperature, employing 1 × solutions of anode (Beyotime, Cat. No. P0752) and cathode running buffers (Beyotime, Cat. No. P0751). The gel was stained with Coomassie brilliant blue R250 for 2 h, and then de‐stained for 4–6 h. A low‐range protein marker (Yeasen, Cat. No. 20344ES72) was used. The absorption spectra of the purified *Ds*RC‐LH1 complex were measured at room temperature by an IMPLEN NP80 Nanophotometer with a wavelength ranging from 300 nm to 900 nm. The sample was loaded in IMPLEN DC 10 disposable cuvettes and the path length was 1.0 mm. The thermal stability of the *Ds*RC‐LH1 complexes purified in salt‐free, Mg^2+^, Ca^2+^, and EDTA‐2Na solutions was assessed via the relative LH1 Q_y_ peak intensities monitored after incubation at 50 °C for 0–90 min.^[^ ^34^ ^]^

### Negative Staining Analysis of DsRC‐LH1s

The EM images were recorded on a Tecnai G2 spirit 120 kV transmission electron microscope (Thermo FEI) using the *Ds*RC‐LH1 samples collected after the SDGC as shown in Figure 4A. For ease of observation, the *Ds*RC‐LH1s were diluted at different multiples with sucrose‐free buffers. In detail, the *Ds*RC‐LH1s purified with salt‐free (control)‐ and EDTA‐2Na‐ solutions were not diluted, and the *Ds*RC‐LH1s purified with Mg^2+^‐ and Ca^2+^‐ solutions were diluted three‐fold, respectively. The EM samples were prepared by a routine procedure using 2% uranium acetate.

### Cryo‐EM Sample Preparation and Data Collection

The concentrations of the *Ds*RC‐LH1 samples were adjusted to an absorbance of ≈0.2 at 867 nm, and all the vitrified samples were prepared following the same protocol. In detail, an aliquot of 2.5 µL purified *Ds*RC‐LH1 complex sample with an absorbance ≈0.2 at 867 nm was applied to a holey carbon grid covered with graphene‐oxide (Quantifoil R1.2/1.3, Au, 300 mesh) and after 60 s, the grids were blotted for 6.5 s at a humidity of 100% and 8 °C, and plunge‐frozen in liquid ethane using a Vitrobot Mark IV (Thermo Fisher Scientific). Cryo‐EM images of the *Ds*RC‐LH1 complexes were recorded with an FEI Titan Krios electron microscope operated at 300 kV with a nominal magnification of 130 000, corresponding to 0.93 Å per pixel at the specimen level for the *Ds*RC‐LH1 complexes purified in salt‐free and Mg^2+^ solutions, and with a nominal magnification of 105 000, corresponding to 1.2 Å per pixel at the specimen level for the *Ds*RC‐LH1 complexes purified in EDTA‐2Na solution. Automated data acquisition was performed using the EPU software in version 3.6 (Thermo Fisher Scientific). The microscope was carefully aligned before data collection to minimize the effects of beam tilt. The dose rate of the electron beam was set to ≈8 e^−^ s^−1^ per physical pixel, and movies were recorded on a Gatan K2 summit camera at four frames/s for 10 s using the super‐resolution mode with a total dose of ≈50 e^−^ Å^−2^ on the specimen. The defocus range was set to −1.5 to −2.5 µm. For the *Ds*RC‐LH1 samples purified in salt‐free, Mg^2+^, and EDTA‐2Na solutions, 10810, 4928, and 6425 movies were recorded, respectively.

### Cryo‐EM Image Processing

All image processing steps were accomplished with cryoSPARC (v4.5.1).^[^ ^35^ ^]^ For the *Ds*RC‐LH1 samples purified in salt‐free solution, a group of 297 movies with clear image contrast were first selected and processed with a pipeline of patch motion correction, patch contrast transfer function (CTF) estimation, exposure curation, particle picking (Blob picker) and 2D classification. Subsequently, 137 364 raw particles were picked and classified into 50 classes, and 24 classes (91338 particles) were selected as the templates to perform further particle picking from 9848 exposures which were obtained from 10810 raw movies according to the same pipeline described above. Totally 9 646 819 particles were picked, 8 172 719 particles were extracted for 2D classification and 2 026 935 particles were selected for ab initio reconstruction. A number of 1 106 552 particles resulting from the ab initio reconstruction were further screened by 3D classification and subsequently used for refinements, including a non‐uniform 3D refinement and a local 3D refinement. The resulting 136533 good particles were refined without any imposed symmetry (*C*1), yielding the final map with a resolution of 2.73 Å according to the gold standard Fourier shell correlation using a criterion of 0.143 (Figure S2, Supporting Information). For the *Ds*RC‐LH1 samples purified in Mg^2+^ and EDTA‐2Na solutions, the data processing procedure was akin to that applied to the *Ds*RC‐LH1 sample purified in salt‐free solutions. Ultimately, two final density maps with resolutions of 2.68 and 2.78 Å (FSC ≥ 0.143) were obtained (Figure S2, Supporting Information).

### Modeling and Refinement

The initial model of the *Ds*RC‐LH1 complex was constructed using the structure of RC‐LH1 from *Rba. sphaeroides* (PDB: 7F0L) as a reference model, and fitting it into the corresponding density map of *Ds*RC‐LH1 using UCSF Chimera.^[^ ^36^ ^]^ The amino acid sequences were then manually mutated to the corresponding sequences of subunits (L, M, H, α, and β) of *Ds*RC‐LH1. The model of the *Ds*RC‐C subunit was developed by employing the structure of the RC‐C subunit from *Rpi. globiformis* (PDB: 7XXF) as a reference model. The remaining fragment was further built manually with COOT.^[^ ^37^ ^]^ The cofactors (BCL, BPH, SPN, HEC, U10, and FE) in the *Ds*RC‐LH1 complex were fitted with corresponding high‐resolution structures from PDB. Models of spheroidenones and modified lipids (phosphatidylglycerols) were generated using the eLBOW tool in Phenix.^[^ ^38^ ^]^ The initial model of the *Ds*RC‐LH1 complex was refined with real‐space refinement against the density map with Phenix.^[^ ^39^ ^]^ The refined model was edited again with COOT to optimize related parameters to a reasonable range.^[^ ^37^ ^]^ The final model was obtained by several iterative operations of these above steps. No CNS constraints were applied during the final model refinement with Phenix. The chimeric model of the *Ds*Cyt *c*_2_‐RC‐C complex was generated using the AlphaFold 3 server.^[^ ^18^ ^]^ The predicted template modeling (pTM) score and the interface predicted template modeling (ipTM) score are 0.71 and 0.69, respectively.^[^ ^29^ ^]^ Figures depicting the models were created with UCSF ChimeraX and PyMOL (Molecular Graphics System, LLC).^[^ ^40^ ^]^ Sequence identity and similarity were estimated by BioEdit (v7.0.5.3), ESPript (v3.0), and MEGA (v11.0.13) tools.^[^ ^41^ ^]^

### Identification of Protein‐O using Mass Spectrum Methods

Mobile phase A (100% water, 0.1% formic acid) and B solution (80% acetonitrile, 0.1% formic acid) were prepared. The lyophilized powder was dissolved in 10 µL of solution A, centrifuged at 14 000 g for 20 min at 4 °C, and 1 µg of the supernatant was injected into a home‐made C18 Nano‐Trap column (Thermo Fisher, 164 535, 5 cm×75 µm, 3 µm). A home‐made analytical column (25 cm × 150 µm, 1.9 µm) was used, the column oven was set as 55 °C, and linear gradient elution was employed for sample elution: 0–3 min, 6% B; 3–53 min, 6–22% B; 53–62 min, 22%–35% B; 62–70 min, 35%–55% B; 70–73 min, 55%–99% B; 73–75 min, 99% B. The separated peptides were analyzed by Orbitrap Eclipse matched with FAIMS (Thermo Fisher), with an ion source of Nanospray Flex (ESI), spray voltage of 2.0 kV, and ion transport capillary temperature of 320 °C. Data‐dependent acquisition mode was adopted by mass spectrometry, FAIMS compensation voltage was set as −45 and −65 respectively and followed to the same acquisition parameters: full scan ranging from m/z 350 to 1500 with a resolution of 60 000 (at m/z 200), an automatic gain control (AGC) target value was 3 × 106 and a maximum ion injection time was Auto. The scan‐round time in MS/MS was set to 1 s, and the precursors in the full scan were selected from high to low abundant and fragmented by higher energy collisional dissociation (HCD), where resolution was 15 000 (at m/z 200), the automatic gain control (AGC) target value was 7.5 × 104, the maximum ion injection time was 22 ms, a normalized collision energy was set as 30%, an intensity threshold was 5.0 × 103, and the dynamic exclusion parameter was 40 s. The raw data of MS detection was named “.raw”.

## Conflict of Interest

The authors declare no conflict of interest.

## Author Contributions

W.W., Y.L., and J.G. contributed equally to this work. J.H.C. and Q.Z. initialized and supervised the project. W.W. and Y.L. cultured the bacteria and purified the sample. W.W., J.G., and S.A. prepared the cryoEM sample and collected the data. J.H.C. processed the data and built atomic models. J.H.C., W.W., Y.L., and J.G. prepared the figures. C.M. and H.G. participated in the sample preparation. J.‐H.C., Q.Z., X.Z., J.‐R.S., and J.T.B. analyzed the data and interpreted the structures. J.‐H.C., Q.Z., X.Z., J.‐R.S., J.T.B., and M.K. wrote the paper.

## Supporting information



Supporting Information

## Data Availability

Atomic coordinates and cryo‐EM map have been deposited in the Protein Data Bank and the Electron Microscopy Data Bank with access numbers: 8YY9 and EMD‐39671 for the *Ds*RC‐LH1 purified in salt‐free solutions, 8YZ2 and EMD‐39683 for the *Ds*RC‐LH1 purified in Mg^2+^ containing solutions, 9JPB and EMD‐61695 for the *Ds*RC‐LH1 purified in EDTA‐2Na solutions, respectively. All other data are presented in the main text or supplementary materials.
