# Supplementary material for: Cryo‐EM Analysis of a Tri‐Heme Cytochrome‐Associated RC‐LH1 Complex from the Marine Photoheterotrophic Bacterium Dinoroseobacter Shibae
Source: Adv Sci (Weinh). 2025 Mar 20;12(18):2413456. doi: 10.1002/advs.202413456 (PMC12079452; doi:10.1002/advs.202413456)
Supplement: Supplementary file 1 — Supporting Information [file ADVS-12-2413456-s001.docx]

Supporting Information

Cryo-EM Analysis of a Tri-Heme Cytochrome-Associated RC-LH1 Complex from the Marine Photoheterotrophic Bacterium *Dinoroseobacter shibae*

Weiwei Wang, Yanting Liu, Jiayi Gu, Shaoya An, Cheng Ma, Haichun Gao, Nianzhi Jiao,

Jian-Ren Shen, J. Thomas Beatty, Michal Koblížek, Xing Zhang,* Qiang Zheng,* and Jing-Hua Chen*

Supplementary Table S1-S4

Supplementary Figures S1-S21

Table S1. Cryo-EM data collection, refinement and validation statistics.

|  | | *Ds*RC-LH1(salt-free) | *Ds*RC-LH1(Mg^2+^) MgCl_2_) | *Ds*RC-LH1(EDTA) |
| --- | --- | --- | --- | --- |
| Magnification | | 130,000 | 130,000 | 105,000 |
| Voltage (kV) | | 300 | 300 | 300 |
| Electron exposure (e^-^/A^2^) | | 50 | 50 | 50 |
| Defocus range (um) | | -1.5~2.5 | -1.5~2.5 | -1.5~2.5 |
| Pixel size (Å) | | 0.93 | 0.93 | 1.2 |
| Symmetry imposed | | *C*1 | *C*1 | *C*1 |
| Micrographs | | 10,810 | 4928 | 6425 |
| Initial particle images (no.) | | 9,646,819 | 4,782,692 | 6,165,146 |
| Final particle images (no.) | | 268,430 | 194,156 | 230,375 |
| Map resolution (Å) (global) | | 2.73 | 2.68 | 2.78 |
| FSC threshold | | 0.143 | 0.143 | 0.143 |
| Map-sharpening B-factor (Å^2^) | | -93 | -93 | -93 |
| Map resolution range (Å) | | 2.5-3.5 | 2.5-3.5 | 2.5-3.5 |
|  | **Refinement** | | | |
| Initial model used (PDB code) | | 6ET5, 5YQ7, 7F0L | 6ET5, 5YQ7, 7F0L | 6ET5, 5YQ7, 7F0L |
| Model resolution | | 2.72 | 2.69 | 2.80 |
| FSC threshold | | 0.143 | 0.143 | 0.143 |
| Model composition | |  |  |  |
| Non-hydrogen atoms | | 28258 | 28258 | 28258 |
| Protein residues | | 2863 | 2863 | 2863 |
| Ligands | | 100 | 100 | 100 |
| B factors (Å^2^) | |  |  |  |
| Protein | | 103.27 | 92.23 | 78.95 |
| Ligand | | 103.89 | 90.58 | 77.62 |
| R.m.s.deviations (PHENIX) | |  |  |  |
| Bond length (Å) | | 0.003 | 0.003 | 0.003 |
| Bond angles (°) | | 0.645 | 0.677 | 0.645 |
| Validation | |  |  |  |
| MolProbity score | | 1.62 | 1.57 | 1.50 |
| Clashscore | | 11.39 | 11.35 | 9.55 |
| Poor rotamer (%) | | 0 | 0 | 0.04 |
| Ramachandran plot | |  |  |  |
| Favored (%) | | 97.78 | 98.28 | 98.10 |
| Allowed (%) | | 2.18 | 1.62 | 1.90 |
| Disallowed (%) | | 0.04 | 0.11 | 0.00 |

**Table S2.** Peptide analysis of the *Ds*RC**-**LH1 complex by mass spectrometry.

| Subunit | Accession | Description | Score | Coverage |
| --- | --- | --- | --- | --- |
| RC-L  (279 a.a.) | ABV95256.1 | Reaction center protein L chain [*Dinoroseobacter shibae* DFL 12 = DSM 16493] | 27.51 | 11% |
| RC-M  (330 a.a.) | ABV95257.1 | Reaction center protein M chain [*Dinoroseobacter shibae* DFL 12 = DSM 16493] | 53.66 | 23% |
| RC-C  (360 a.a.) | ABV95258.1 | photosynthetic reaction center cytochrome *c* subunit precursor [*Dinoroseobacter shibae* DFL 12 = DSM 16493] | 114.26 | 56% |
| RC-H  (256 a.a.) | ABV95273.1 | Reaction center protein H chain [*Dinoroseobacter shibae* DFL 12 = DSM 16493] | 471.52 | 70% |
| LH-α  (53 a.a.) | ABV95255.1 | Antenna pigment protein alpha chain [*Dinoroseobacter shibae* DFL 12 = DSM 16493] | 31.66 | 23% |
| LH-β  (49 a.a.) | ABV95254.1 | Antenna pigment protein beta chain [*Dinoroseobacter shibae* DFL 12 = DSM 16493] | 4.17 | 59% |
| Protein-O  (239 a.a.) | ABV93056.1 | Uncharacterized protein  [*Dinoroseobacter shibae* DFL 12 = DSM 16493] | 10.22 | 12% |

The length of each protein subunit is indicated in the parentheses.

**Table S3.** Protein Blast analysis of protein-O using UniProt.

| Entry | Protein name | Organism | Length (a.a.) | Identity | E-values |
| --- | --- | --- | --- | --- | --- |
| A8LIU2_DINSH | Protein-O | *Dinoroseobacter shibae*  DFL 12 | 239 | 100% | 1.7e-173 |
| A0A1B6YTT7_9RHOB | ATP synthase subunit E | *Rhodobacteraceae bacterium* EhC02 | 243 | 49.6% | 3.6e-73 |
| A0A0B4BWW1_9RHOB | ATP synthase subunit E | *Tateyamaria* sp. ANG-S1 | 252 | 47% | 5.9e-69 |
| A0A369TQH2_9RHOB | Endonuclease | *Thalassococcus profundi* | 261 | 44.4% | 1.9e-64 |
| A0A0W7WEX8_9RHOB | ATP synthase subunit E | *Pseudoponticoccus marisrubri* | 279 | 45% | 5.3e-63 |
| A0A099TAF4_9RHOB | ATP synthase subunit E | *Thalassobacter* sp.  16PALIMAR09 | 237 | 44% | 4.8e-62 |
| A0A1X6Z6V7_9RHOB | NADH dehydrogenase subunit E | *Roseivivax jejudonensis* | 260 | 42.3% | 9.6e-62 |
| A0A0T5P8C4_9RHOB | NADH dehydrogenase subunit E | *Roseovarius indicus* | 247 | 43.3% | 7.4e-61 |
| A0A4Q1ZZC2_9RHOB | NADH:ubiquinone oxidoreductase | *Roseovarius* sp. A46 | 239 | 46.1% | 2.3e-60 |

Only listed the proteins with scores over 500.

**Table S4. Phototrophic bacteria used for the sequence alignment of L and M subunits.**

|  | Organisms | L subunit | M subunit |
| --- | --- | --- | --- |
| 1 | *Dinoroseobacter shibae* DFL 12 | WP_012180179.1 | WP_012180180.1 |
| 2 | *Roseobater denitrificans* OCH114 | WP_011566461.1 | WP_011566460.1 |
| 3 | *Roseobacter* sp. AzwK-3b | WP_007814241.1 | WP_007814239.1 |
| 4 | *Roseivivax halotolerans* NBRC16686 | WP_093009161.1 | WP_037259284.1 |
| 5 | *Roseovarius* sp. 217 | WP_009819266.1 | WP_009819265.1 |
| 6 | *Jannaschia* sp. CCS1 | WP_011453299.1 | WP_011453298.1 |
| 7 | *Yoonia vestfoldensis_*SKA53 | WP_007206415.1 | WP_040483288.1 |
| 8 | *Citromicrobium* sp. JL477 | ABG90863.1 | WP_010236075.1 |
| 9 | *Erythrobacter litoralis* | WP_034904389.1 | WP_034904391.1 |
| 10 | *Erythrobacter* sp. NAP1 | WP_007164991.1 | WP_007164992.1 |
| 11 | *Congregibacter litoralis* | WP_023660413.1 | WP_008296327.1 |
| 12 | *Rhodobacter sphaeroides* 2.4.1 | WP_002720421.1 | WP_002720420.1 |
| 13 | *Rhodopseudomonas palustris* | WP_011157088.1 | CAE26970.1 |
| 14 | *Rhodobacter capsulatus* SB 1003 | WP_013066437.1 | WP_013066438.1 |
| 15 | *Rhodopila globiformis* | PPQ33045.1 | PPQ33044.1 |
| 16 | *Rhodobacter veldkampii* | PTE17763.1 | PTE17764.1 |
| 17 | *Blastochloris viridis* | CAA27550.1 | WP_055036367.1 |
| 18 | *Blastomonas aquatica* | GGB75251.1 | GGB75264.1 |
| 19 | *Thiorhodovibrio* strain 970 | AEM00417.1 | AEM00418.1 |
| 20 | *Gemmatimonas phototrophica* | WP_026850327.1 | WP_082821114.1 |
| 21 | *Thermochromatium tepidum* | BAI67782.1 | BAF80146.2 |
| 22 | *Roseiflexus castenholzii* | BAC76414.1 | WP_012122217.1 |
| 23 | *Chloroflexus aurantiacus* | WP_012256940.1 | WP_012256939.1 |

**
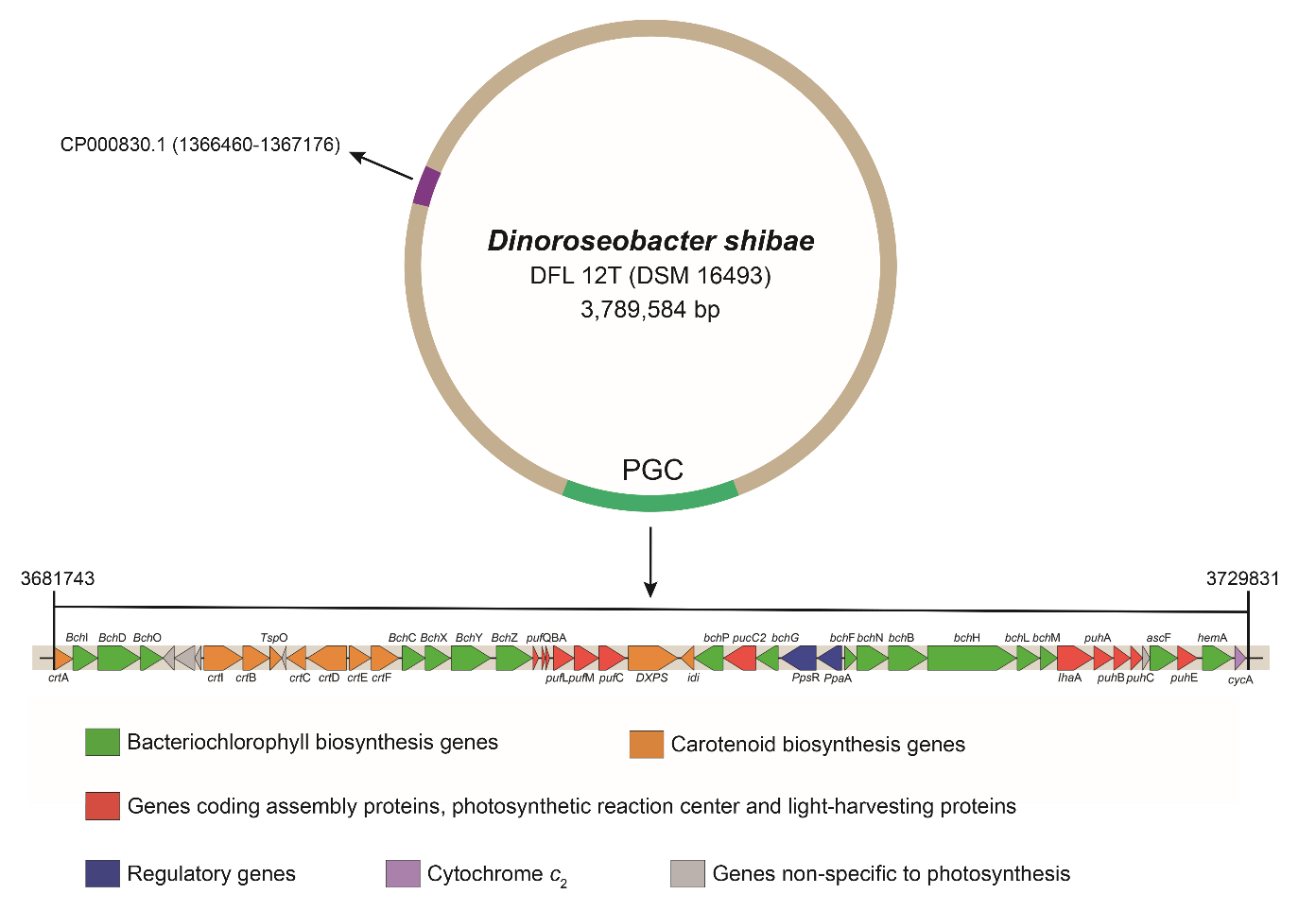
**

**Figure S1. The photosynthetic gene cluster (PGC) and location of the protein-O gene in the chromosome of *D. shibae* DFL12^T^.** Genes associated with different functions are colored differently. The nucleic acid segment CP000830.1(1366460-1367176) encodes protein-O in the *Ds*RC-LH1.

**
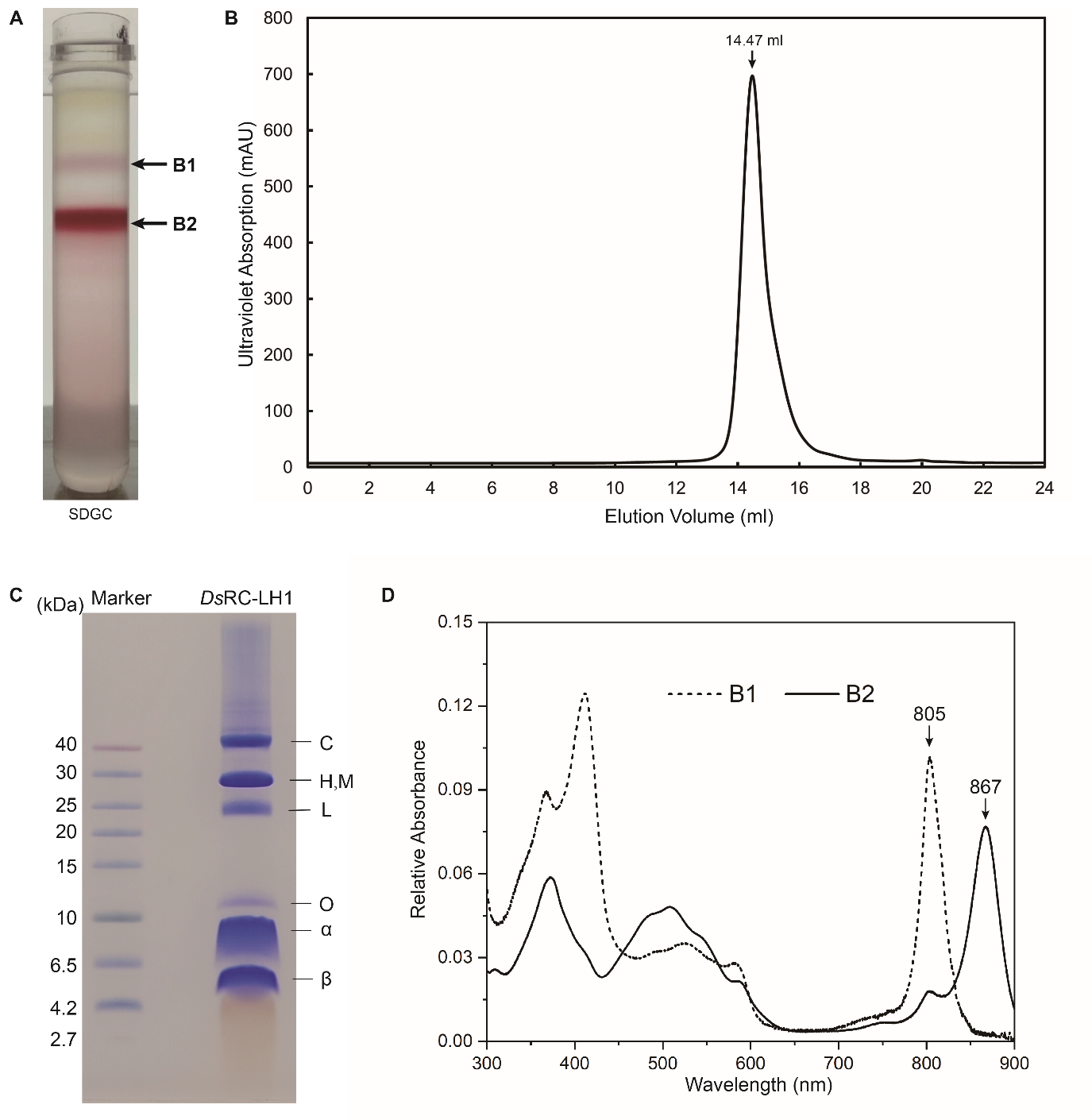
**

**Figure S2. Purification and biochemical characterization of *Ds*RC-LH1.** (A) Sucrose density gradient centrifugation (SDGC) for purification of *Ds*RC-LH1. Band B2 was collected for further experiments. (B) Gel filtration analysis of *Ds*RC-LH1 with a Superose 6 Increase column (GE). (C) Tricine-SDS-PAGE analysis of the purified *Ds*RC-LH1 complex. Each band was cut and collected for mass spectrometric analysis. The bands corresponding to the L and M subunits were fused together and could not be separated. The protein-O band showed a smaller molecular weight than its predicted size. (D) Room-temperature absorption spectra of the two bands (B1 and B2) in SDGC. The B1 (dotted line) and B2 (solid line) bands exhibited maximum Q_y_ absorptions at 805 and 867 nm, respectively. The spectra were normalized at 651 nm.

**
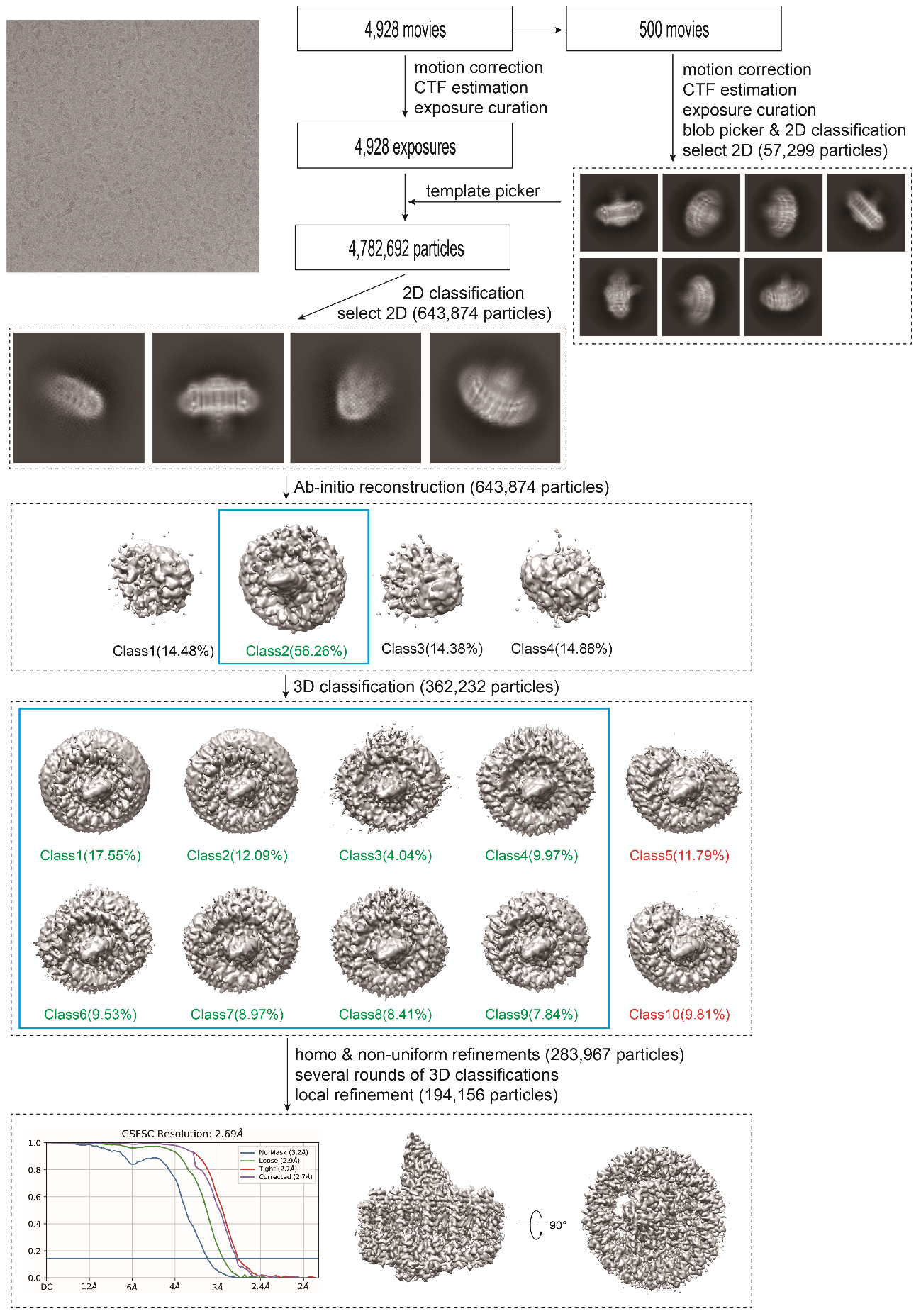
**

**Figure S3.** **Procedure for cryo-EM data processing of the *Ds*RC-LH1 complex purified with Mg^2+^**-**containing buffers**. Particles in the light blue boxes were used for subsequent processing.


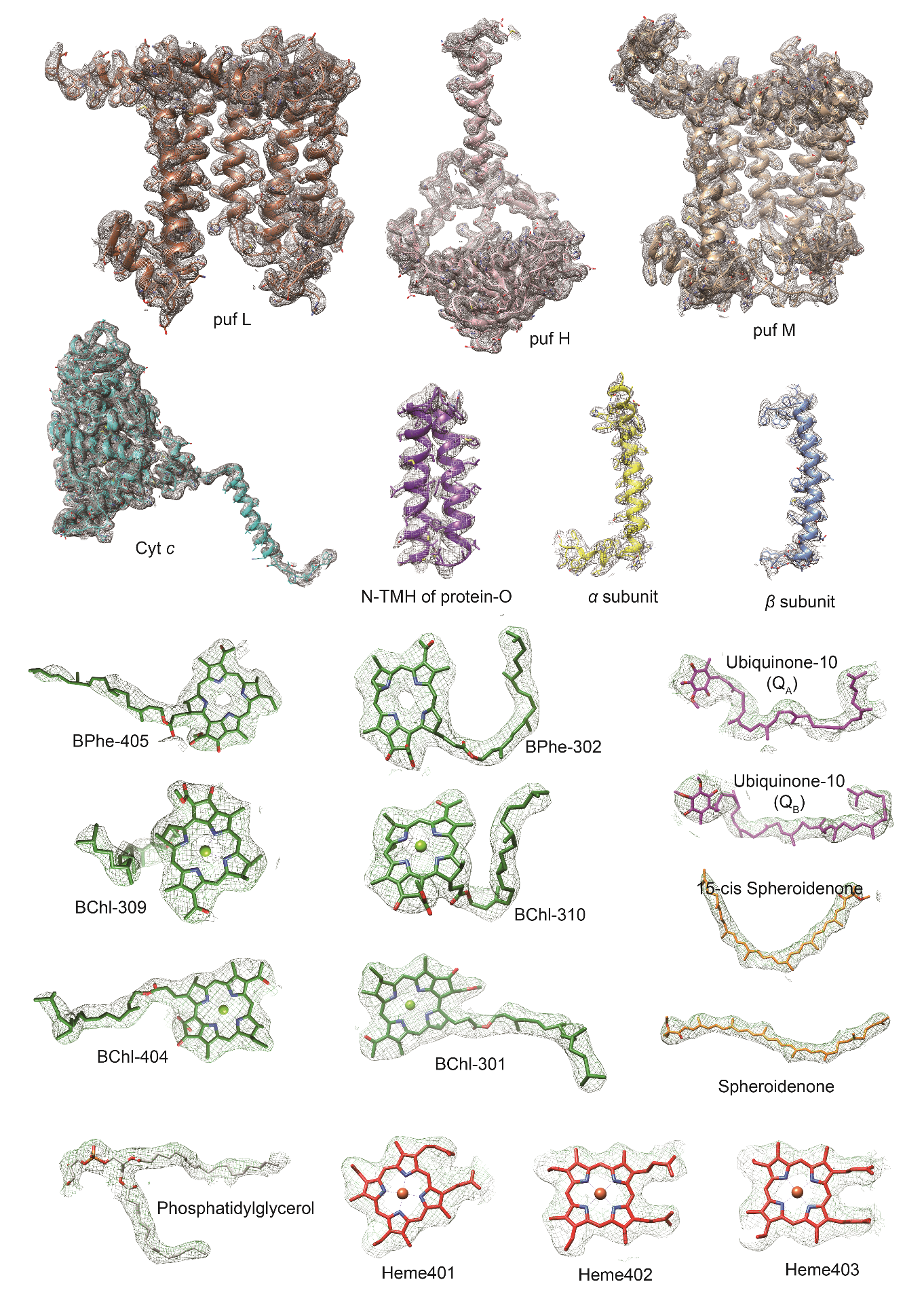


**Figure S4.** **Cryo-EM densities of typical structural elements and cofactors of the *Ds*RC-LH1.** The electron density is shown as gray mesh, and the structural elements and cofactors are displayed as sticks with the same colors as shown in Figure 1.


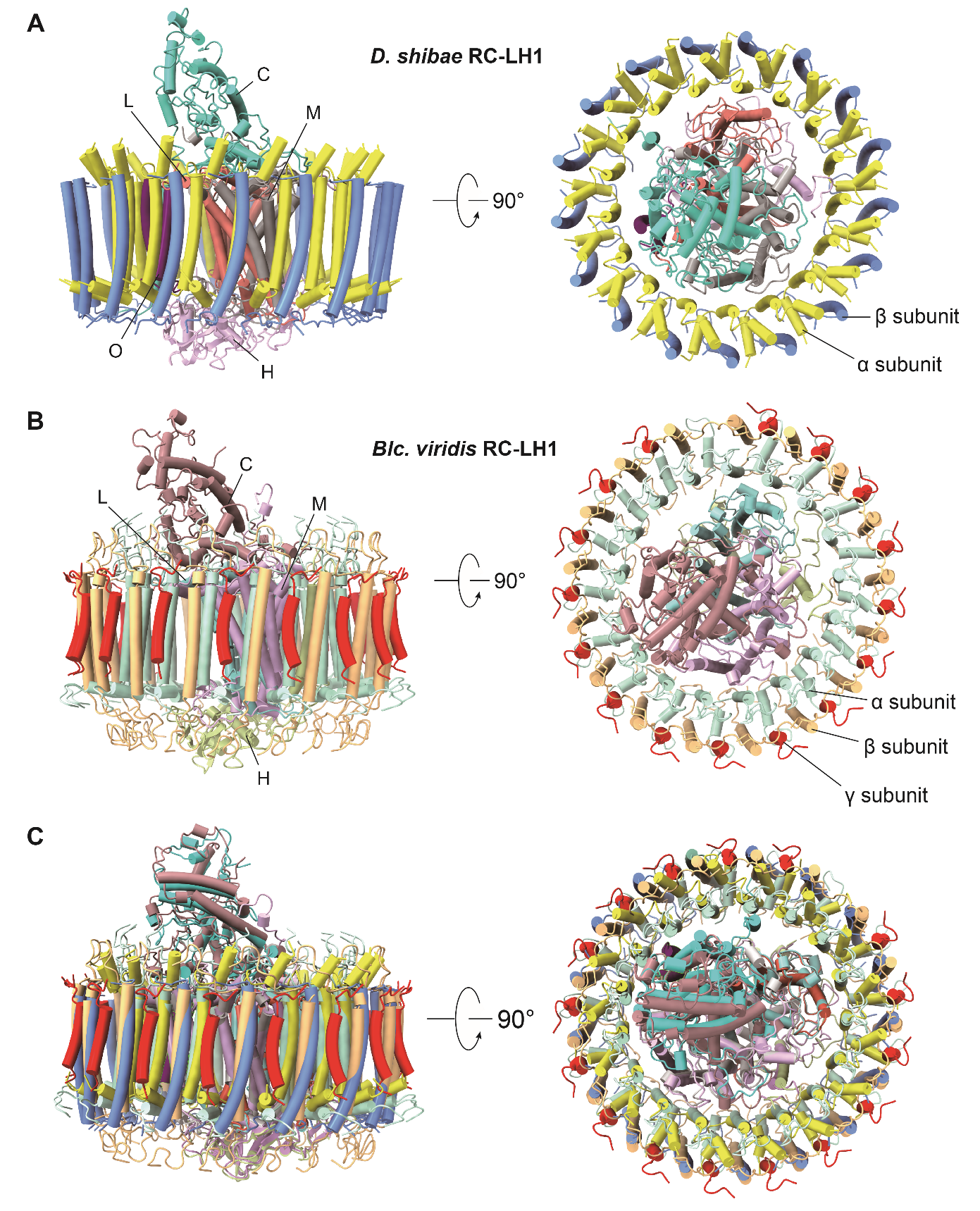


**Figure S5.** **Comparison of the** **RC-LH1 structures from *D. shibae* and *Blc. viridis*.** (A) Structure of *Ds*RC-LH1 shown in cartoon mode in side and top (periplasm) views. The LH ring comprises 17 heterodimers of α and β subunits. (B) Structure of *Blc. viridis* RC-LH1 shown in cartoon mode in side and top (periplasm) views. The LH ring comprises 17 heterodimers of α and β subunits, and 16 additional polypeptides (γ subunits) located in the intervals between adjacent β subunits. (C) Superposition of RC-LH1 structures from *D. shibae* and *Blc. viridis* in side and top (periplasm) views, respectively.


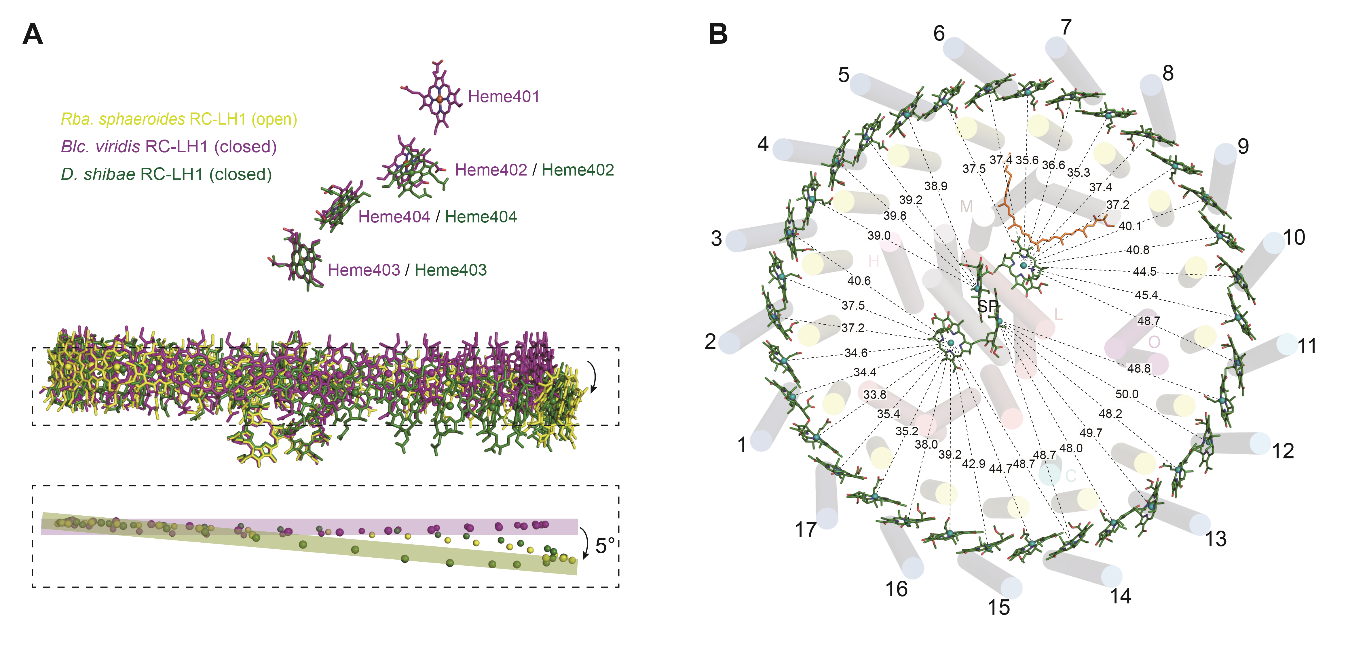


**Figure S6. Distribution of LH BChls within the *Ds*RC-LH1 and alignment of hemes from *Ds*RC-C with *Blc. viridis* RC-C hemes.** (A) Arrangement of the LH BChls of *Ds*RC-LH1 (dark green) and their superposition with those of the *Rba. sphaeroides* RC-LH1 (yellow) and *Blc. viridis* RC-LH1 (purple) in a side view. When fixing the subunit L of *Ds*RC, *Rba. sphaeroides* RC and *Blc. viridis* RC, the Mg atoms of *Ds*LH1 are arranged in a plane which overlaps with that of *Rba. sphaeroides* LH1 but tilts five degrees relative to that of *Blc. viridis* LH1. (B) Arrangement of the BChls within the *Ds*RC-LH1 complex viewed from the periplasmic side. The center-to-center distances between BChls of the RC and the LH1 ring are indicated.

**
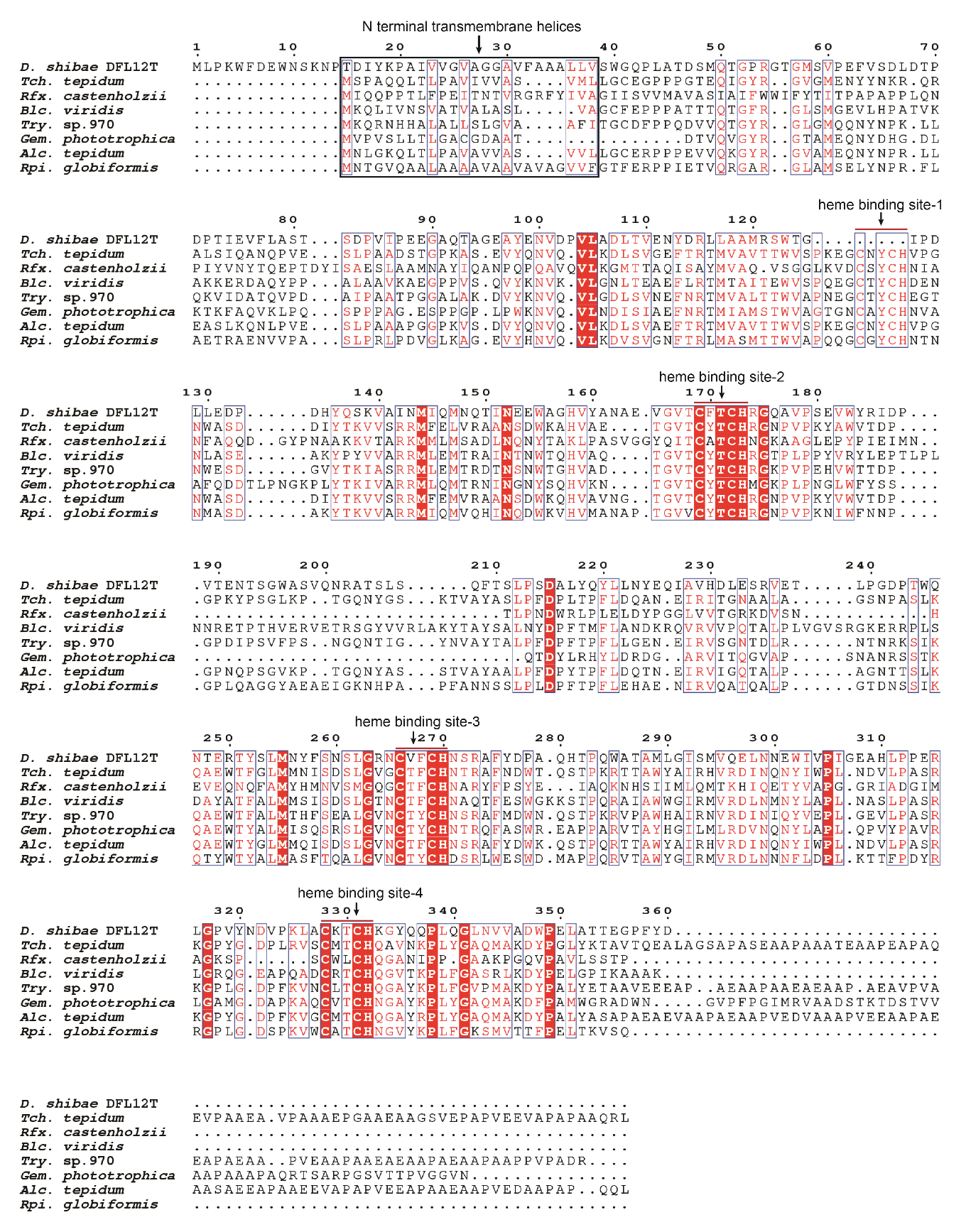
**

**Figure S7.** Multiple sequence alignment of *Ds*RC-C and RC-C subunits from purple bacteria. The black box indicates the N**-**terminal transmembrane domain of RC-C subunits. The conserved heme**-**binding sites in purple bacterial RC-C subunits are indicated with black arrows and the corresponding residues at the first heme-binding site are missing in *Ds*RC-C.

**
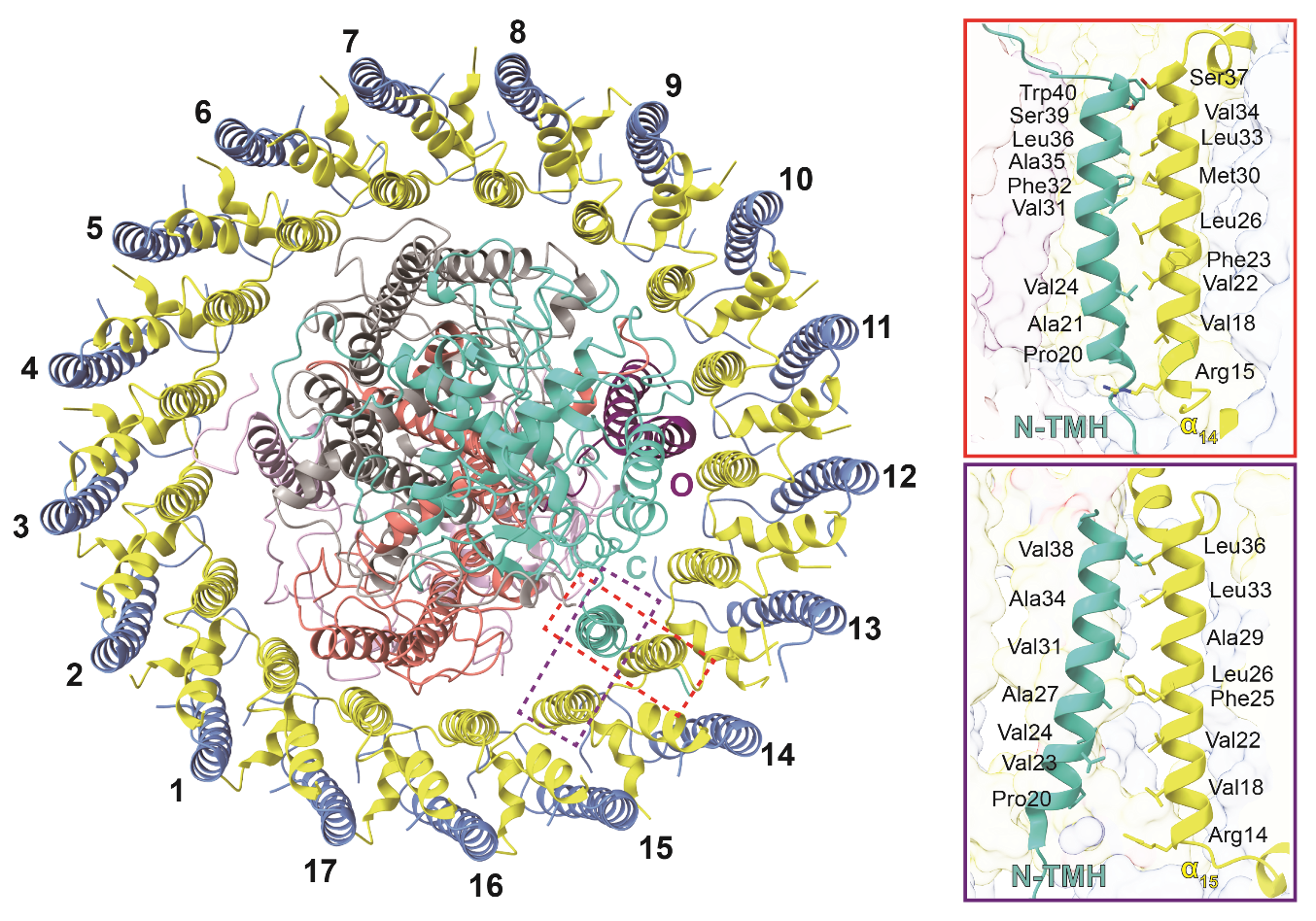
**

**Figure S8.** Interactions between the N-terminal TMH of *Ds*RC-C and its nearby LH α subunits. The red and purple dashed boxes indicate the subunit-pairs of the N**-**terminal TMH of *Ds*RC-C and α_14_, and the N**-**terminal TMH of *Ds*RC-C and α_15_, respectively. The residues at the interface of each subunit**-**pair are depicted in the right panels.

**
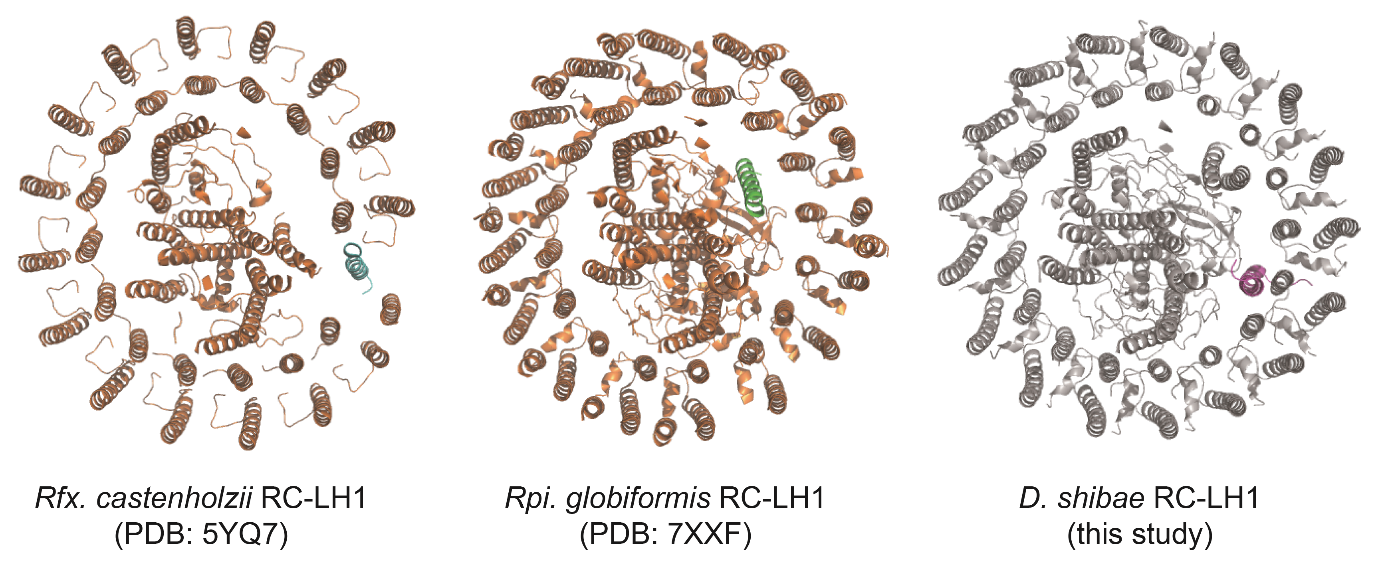
**

**Figure S9.** Locations of the N**-**terminal TMHs of RC-C subunits within RC-LH1s of *Rfx. castenholzii*, *Rpi. globiformis* and *D. shibae*. The soluble domain of each RC-C subunit was cut for clarity. The N**-**terminal TMH of *Rfx. castenholzii* RC-C (colored in cyan) inserts into the LH ring and occupies a position corresponding to a LH α subunit. The N**-**terminal TMH of *Rpi. globiformis* RC-C (colored in green) resides close to the RC core, distant from the LH ring. The N**-**terminal TMH of *Ds*RC-C (colored in purple) is close to one LH α subunit with broad interactions.


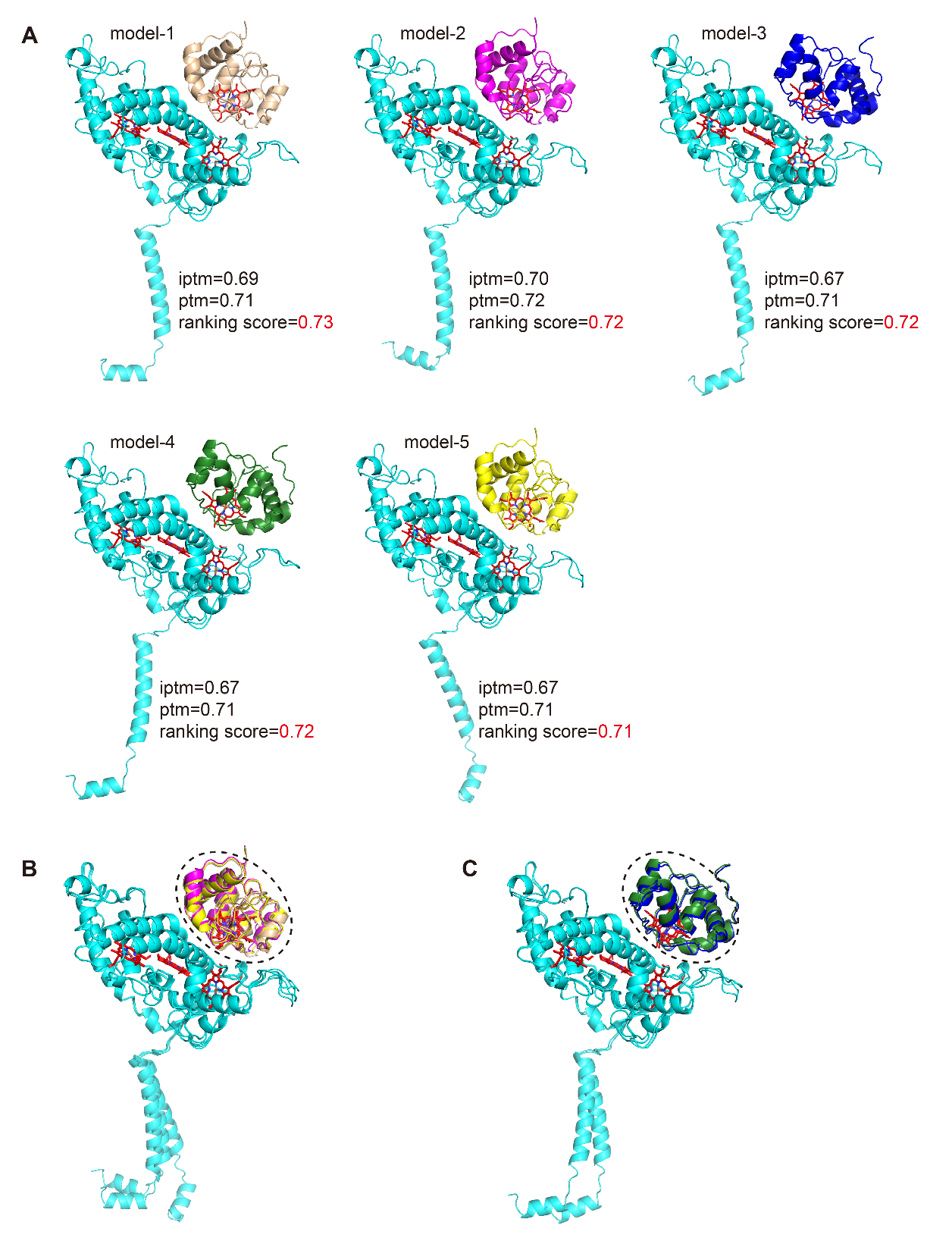


**Figure S10.** Predicted *Ds*Cyt *c*_2_-RC-C models using AlphaFold 3. (A) Structures of five predicted *Ds*Cyt *c*_2_-RC-LH1 models using AlphaFold 3. The confidence metrics of each model are listed next to them. The *Ds*RC-C subunit is colored in cyan, and the *Ds*Cyt *c*_2_ subunits are colored in different colors. (B) Alignment of the structures of the predicted models 1, 2 and 5. (C) Alignment of the structures of the predicted models 3 and 4.


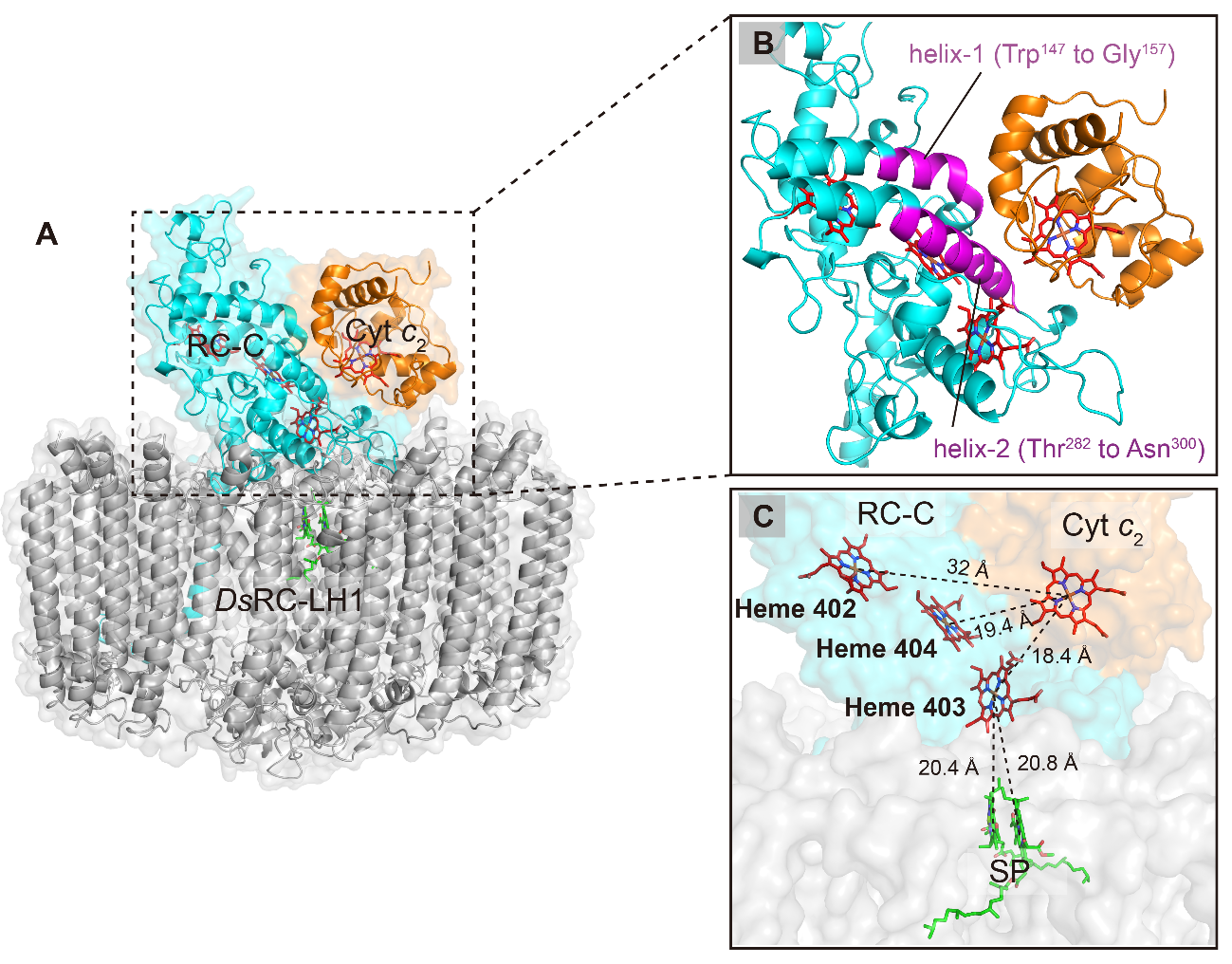


**Figure S11.** Structural analysis of predicted *Ds*Cyt *c*_2_-RC-C model. (A) Proposed model of the *Ds*Cyt *c*_2_-RC-LH1 complex. The *Ds*RC-C and Cyt *c*_2_ subunits are colored in cyan and orange, respectively. (B) The interface of *Ds*RC-C and Cyt *c*_2_ subunits. Cyt *c*_2_ is attached to two helices (from Trp^147^ to Gly^157^ and Thr^282^ to Asn^300^), and a loop (from Ala^274^ to His^281^) between them. (C) The distances between the central iron atoms of the heme groups in Cyt *c*_2_ and *Ds*RC-C, and the iron-magnesium distances between the heme group in *Ds*RC-C and the SP.

**
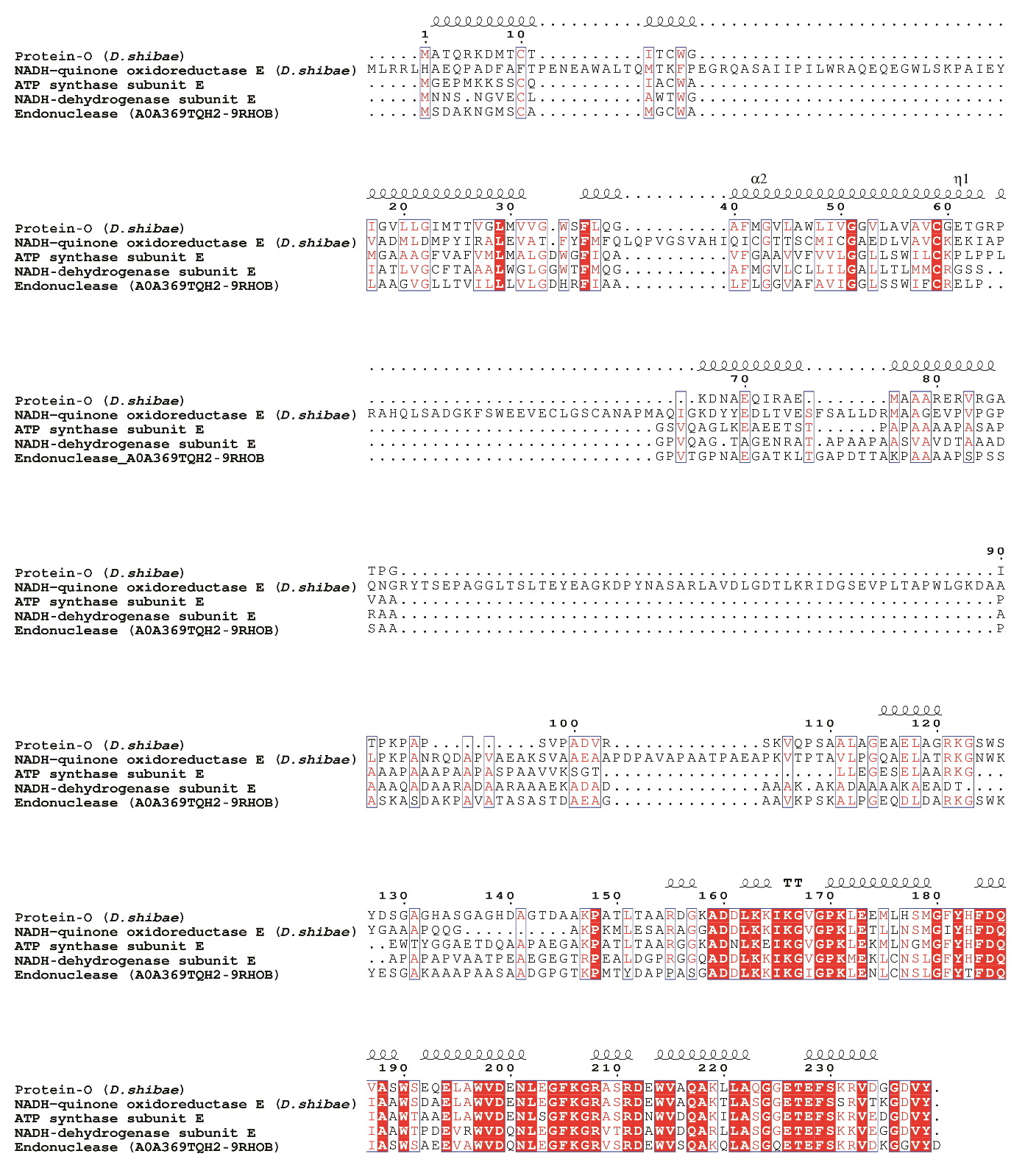
**

**Figure S12.** Sequence alignment of protein-O and NADH-quinone oxidoreductase subunit E, ATP synthase subunit E, NADH-dehydrogenase subunit E and endonuclease.

**
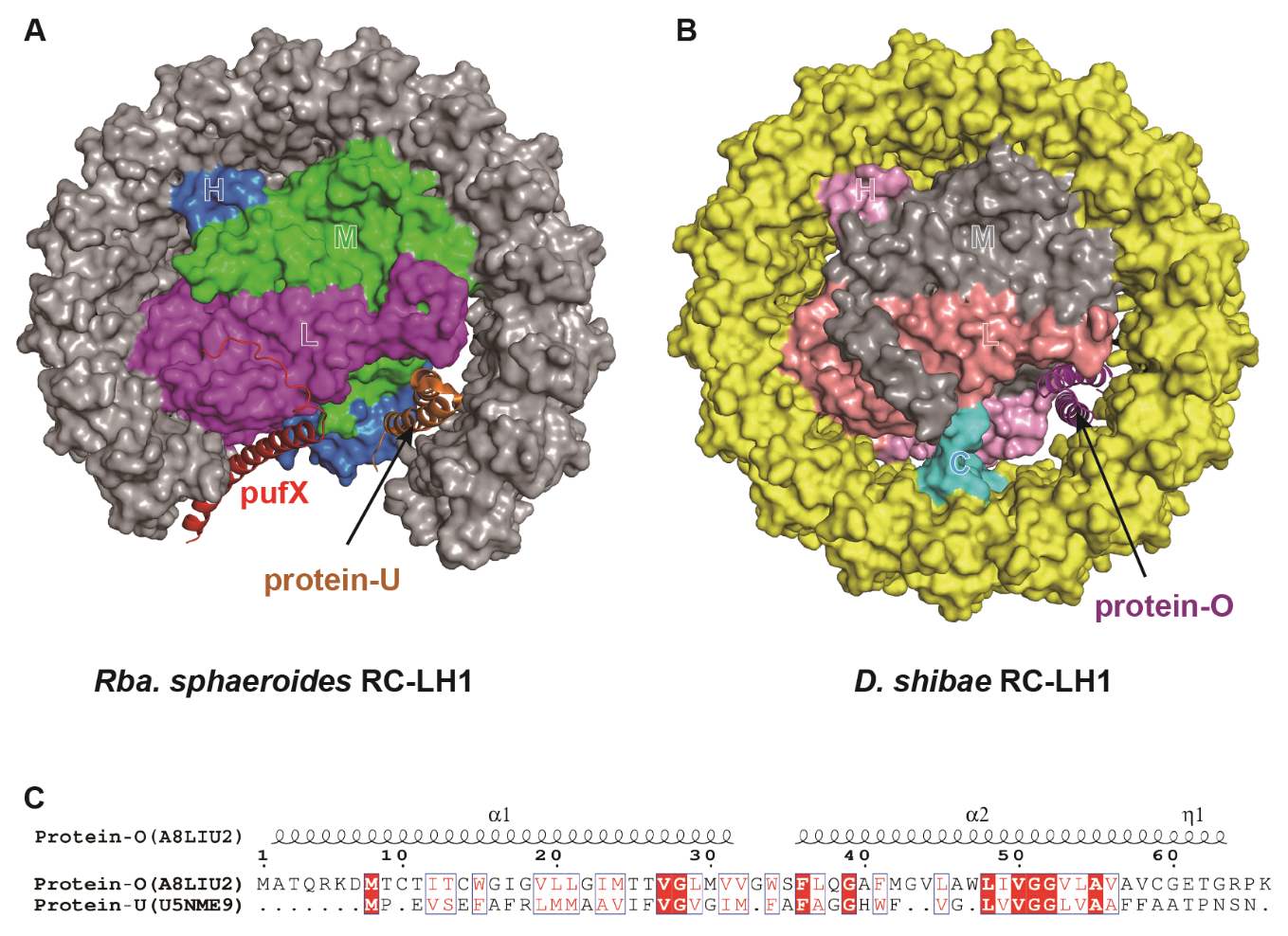
**

**Figure S13.** Comparative analysis of protein-U and protein-O. Both protein-U in the *Rba. sphaeroides* RC**-**LH1 (A) and protein-O in *Ds*RC-LH1 (B) are located at a similar position between the RC**-**LM heterodimer and the LH ring. (C) Sequence alignment of the N**-**terminal TMHs of protein-O and protein-U.

**
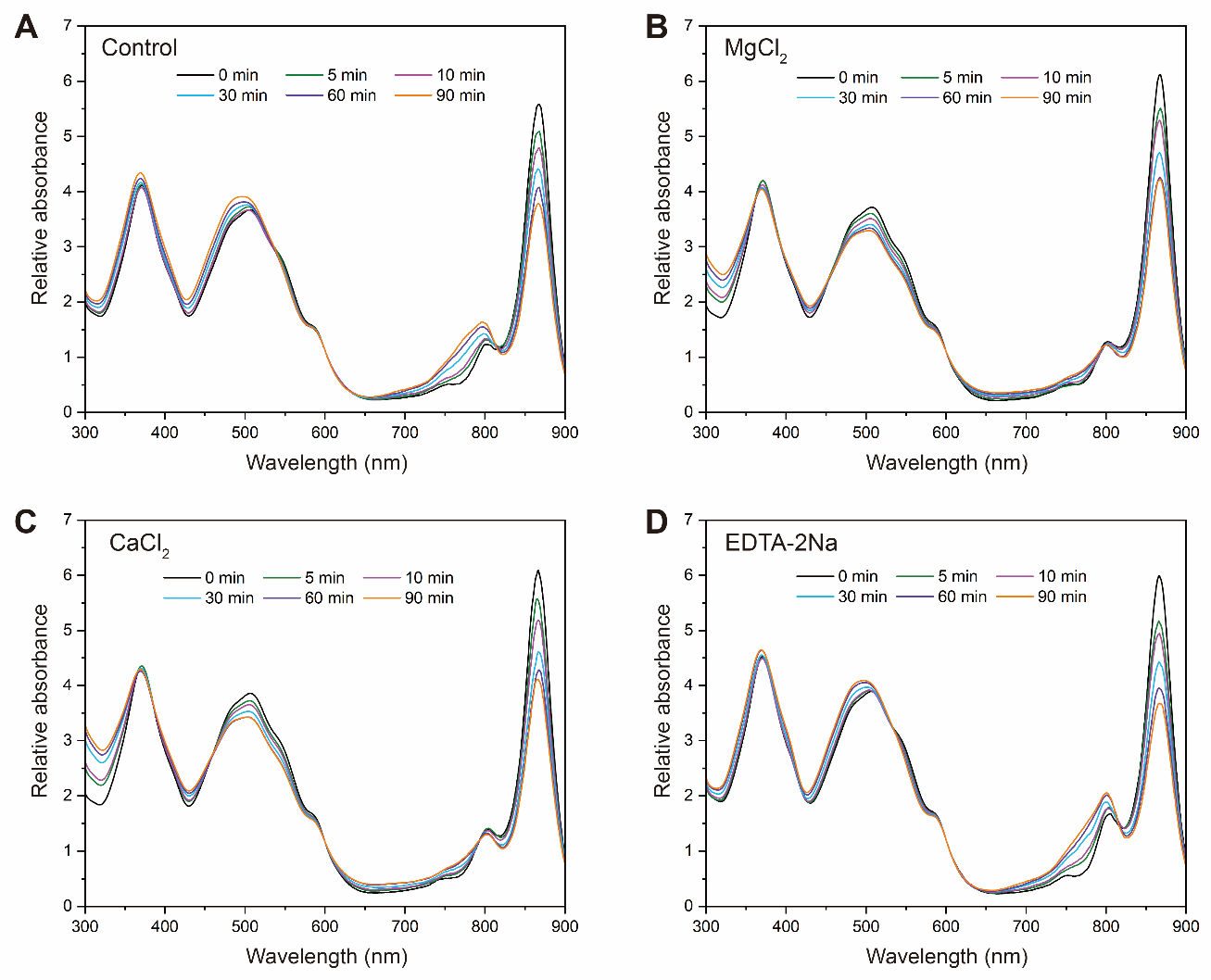
**

Figure S14. Changes of absorption spectra at 50 °C for the *Ds*RC-LH1s as functions of time. The *Ds*RC-LH1 samples were purified in salt-free (control) (A), Mg^2+^ (B), Ca^2+^ (C) and EDTA-2Na (D) solutions. The spectra were normalized at 600 nm.

**
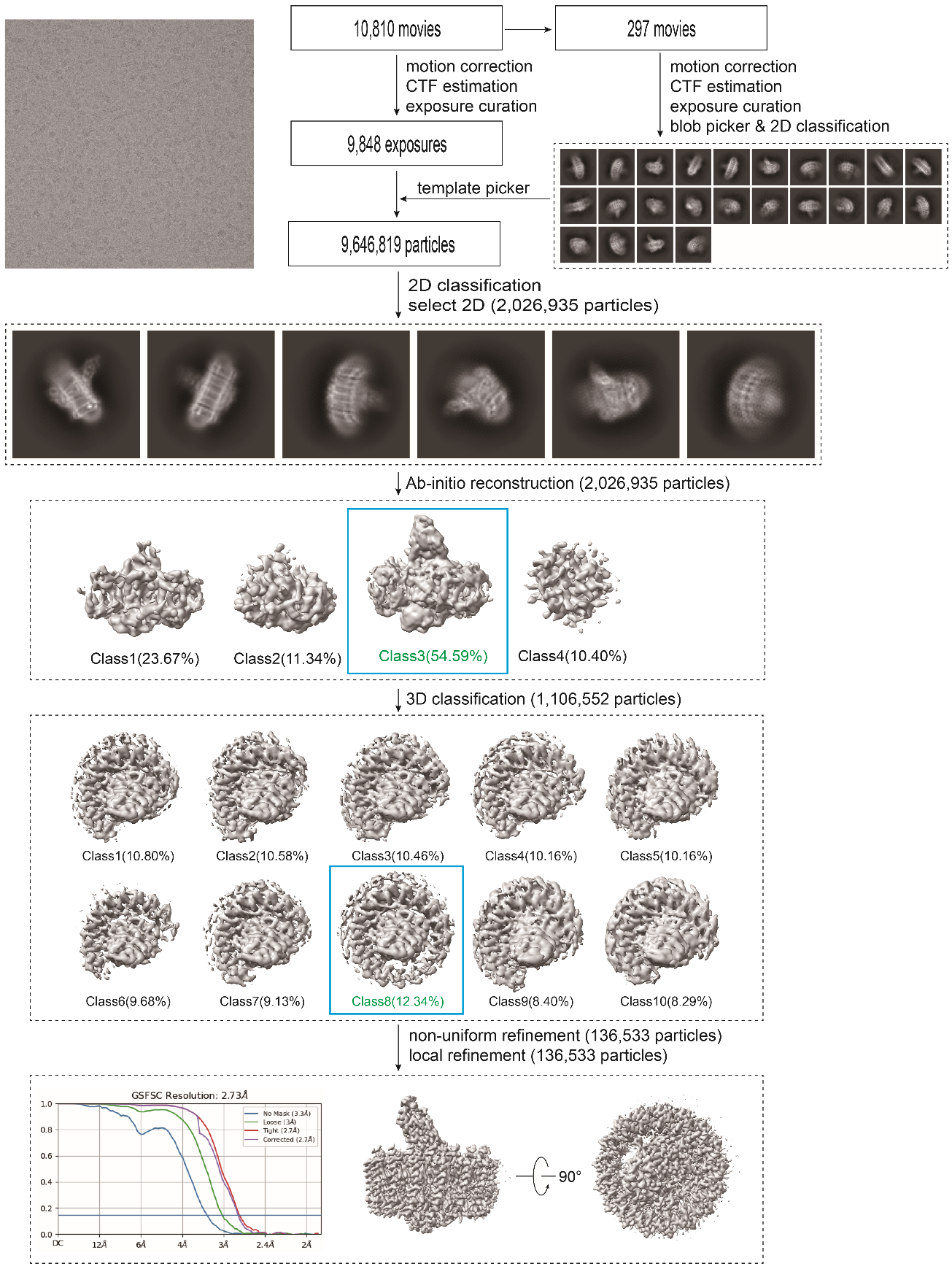
**

**Figure S15.** **The procedure for cryo-EM data processing of the *Ds*RC-LH1 complex purified in salt-free** **buffers**. Particles in the light blue boxes were used for subsequent processing.


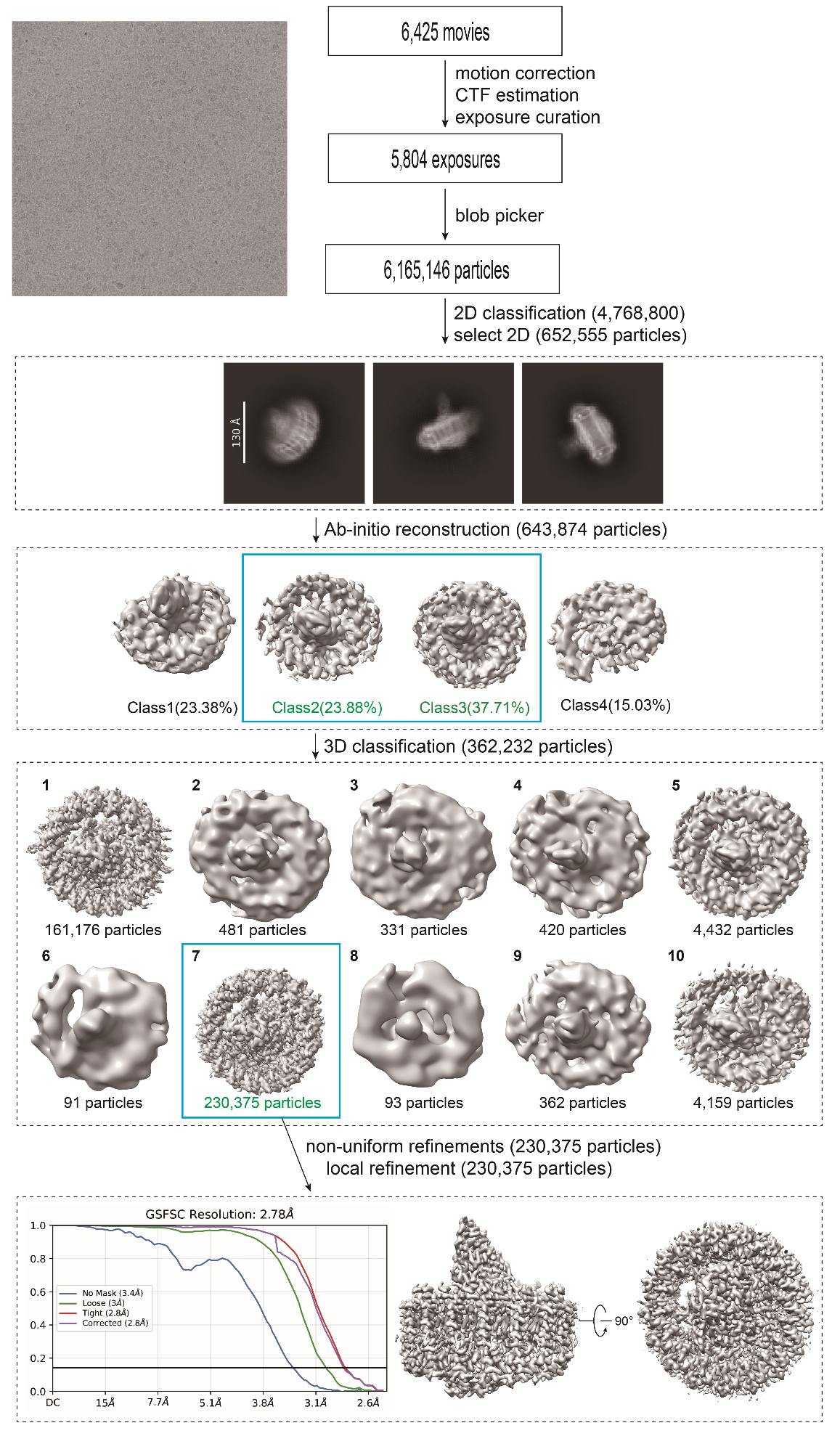


**Figure S16.** **The procedure for cryo-EM data processing of the *Ds*RC-LH1 complex purified in EDTA-2Na containing buffers**. Particles in the light blue boxes were used for subsequent processing.

**
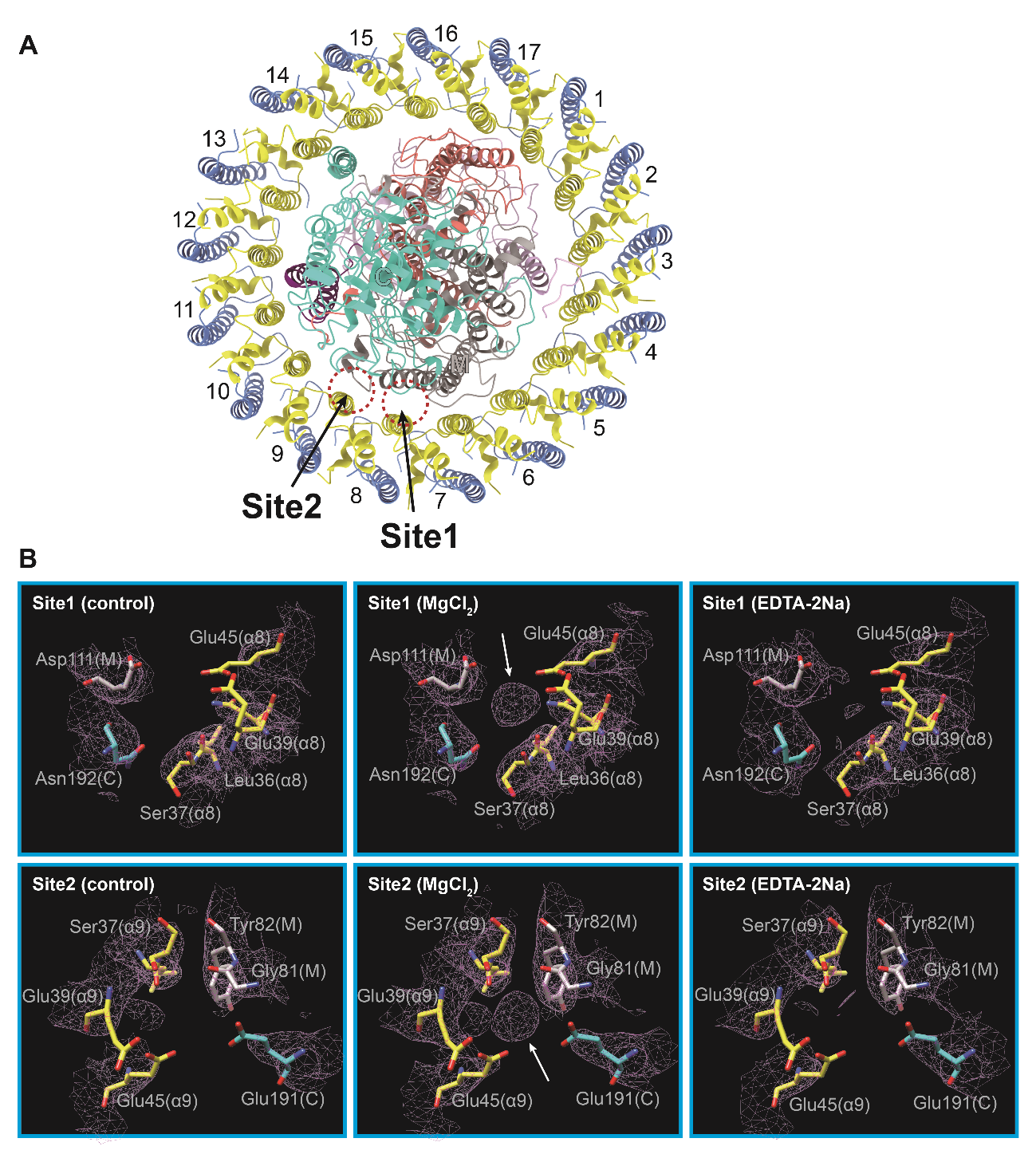
**

**Figure S17.** Local differences at the interfaces between RC and LH1 of ***Ds*RC-LH1s purified in different buffers**. Two additional densities (site1 and site2) (A) which are located at the interfaces between RC-M, RC-C and the 8-9th LH α subunits only in the *Ds*RC-LH1 sample purified using the Mg^2+^ solution (B). The extra density is indicated by white arrows.**
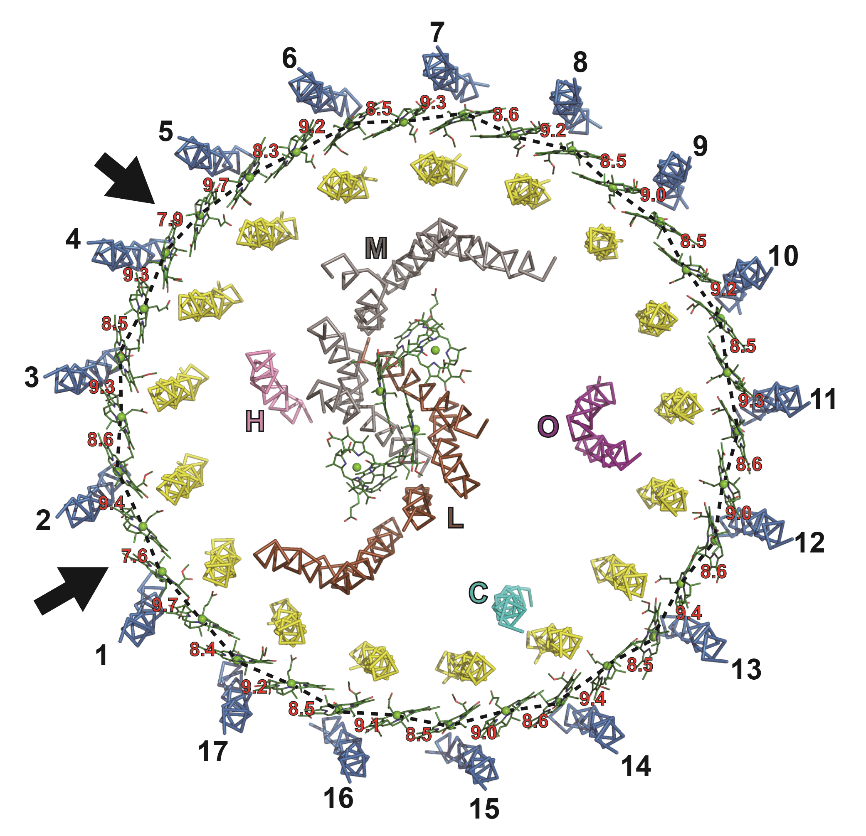
**

**Figure S18.** The Mg-Mg distances between the adjacent LH BChls in *Ds*RC-LH1. The transmembrane domains of protein subunits are shown in cartoon mode with each subunit being colored as in Figure 1. The extra-membrane segment of each subunit is cut for clarity. BChls and BPhes are shown in stick mode and colored in green. The Mg**-**Mg distances between the adjacent LH BChls are indicated by dashed lines. Black arrows indicate the two shortest distances between the adjacent BChls along the *Ds*LH ring.


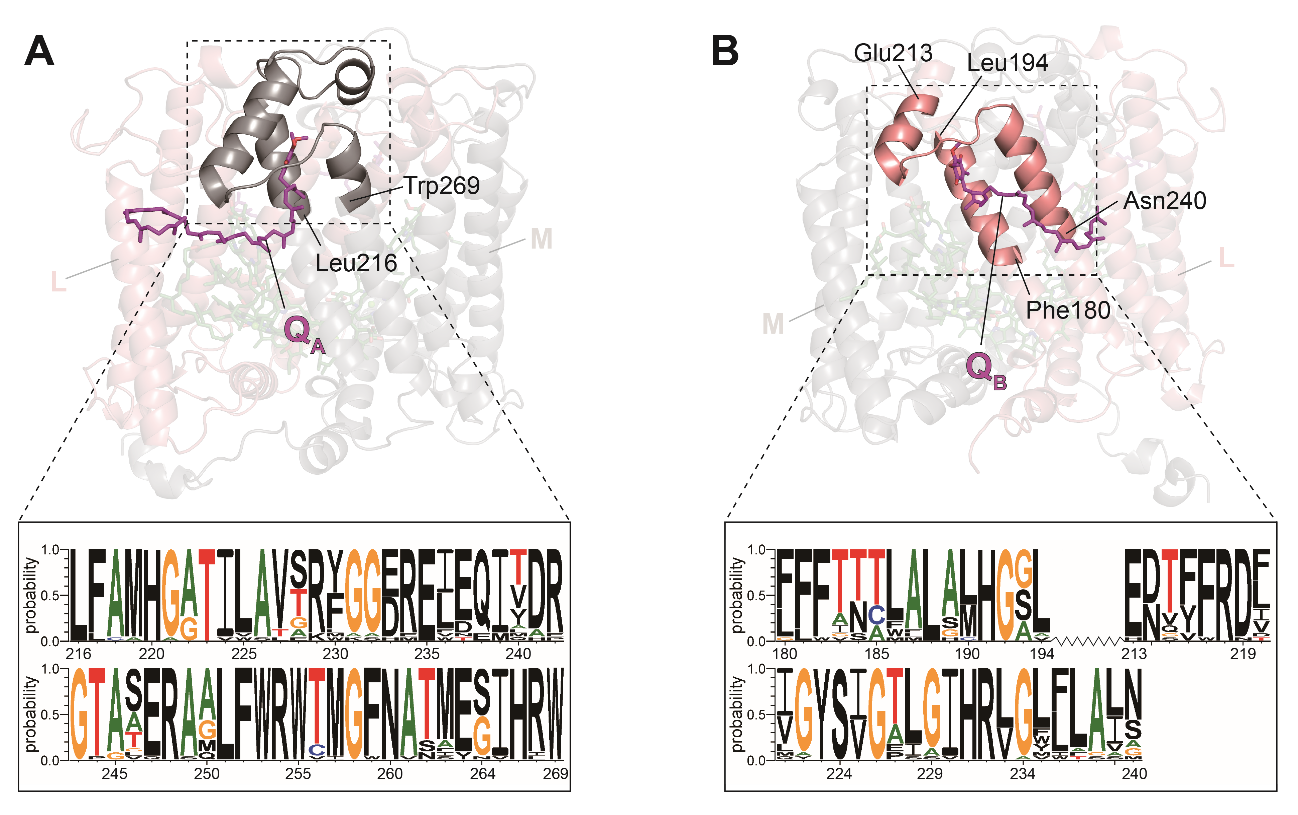


**Figure S19. Surrounding residues of quinones in the *Ds*RC-LH1.** The surrounding residues of Q_A_ and Q_B_ are indicated by dashed boxes. Residues from *Ds*RC-L (A) and *Ds*RC-M (B) are colored in salmon and dark gray, respectively. The sequence logos represent the alignment of residues (Leu^216^ to Trp^269^ from the M subunit, Phe^180^ to Leu^194^ and Glu^213^ to Asn^240^ from the L subunit) from *Ds*RC-LH1s and the corresponding residues from other RC-LH1s in phototrophs shown in Supplementary Table 4.

**
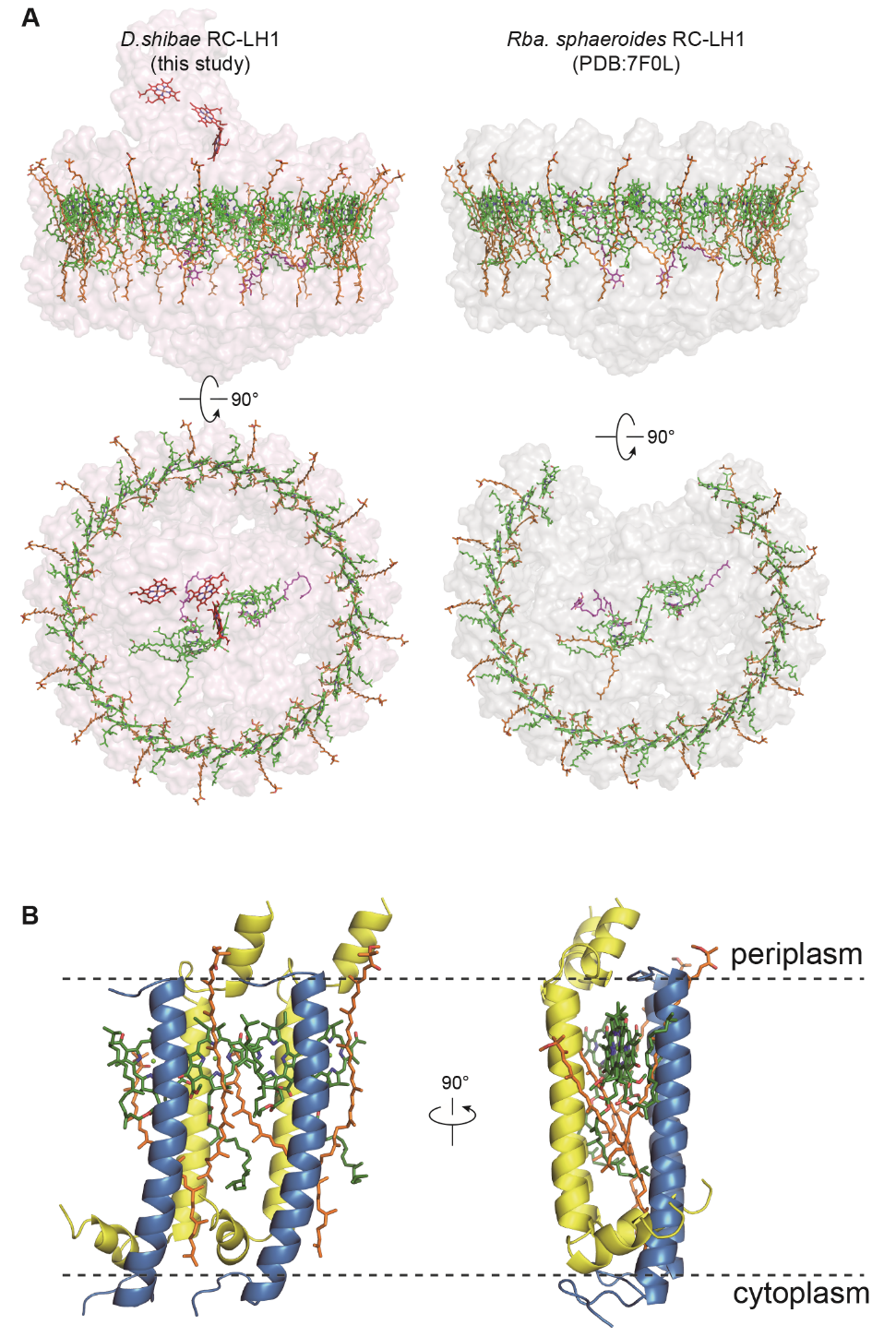
**

**Figure S20.** Comparison of pigment arrangement within RC**-**LH1s of *D. shibae* and *Rba. sphaeroides*. (A) Side and top (periplasmic) view of the pigment distributions within the RC**-**LH1s of *D. shibae* and *Rba.* *sphaeroides*. BChls and carotenoids are colored in green and orange, respectively. The *Ds*LH1 contains 17 αβ heterodimers which jointly form a closed ring. Each *Ds*LH αβ heterodimer harbors two BChls and two carotenoids (B), which is similar to that of *Rba. sphaeroides*. However, the LH rings in *Rba. sphaeroides* are incomplete. The α and β subunits in (B) are shown in cartoon mode, and are colored in yellow and blue, respectively.


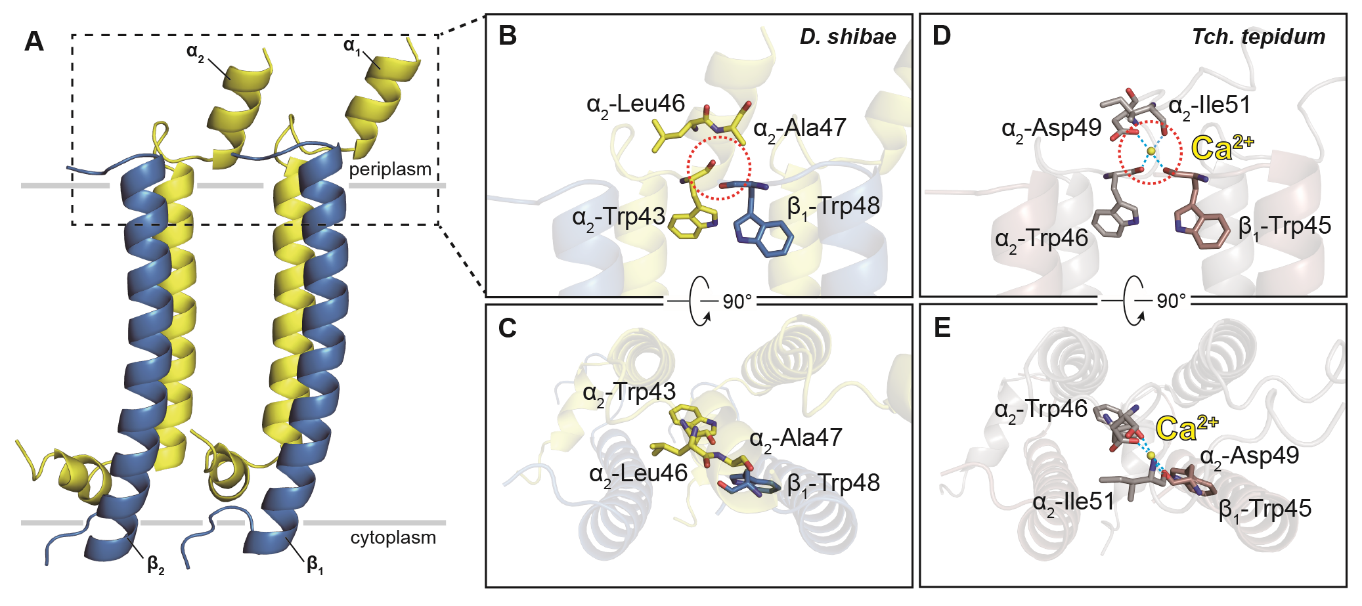


**Figure S21.** Structural analysis of the interfacial environments of adjacent LH αβ heterodimers in the *Ds*RC-LH1 and *Tch. tepidum* RC**-**LH1. (A) Structure of two adjacent *Ds*LH αβ heterodimers (α_1_β_1_ and α_2_β_2_) shown in cartoon mode. (B, C) The interfacial residues of adjacent *Ds*LH αβ heterodimers at the periplasmic side. Hydrogen bonds could not form among these residues because the shortest distance between them exceeds 4 Å. (D, E) Ca^2+^**-**binding site at the interface of adjacent *Tch. tepidum* LH αβ heterodimers at the periplasmic side. The central Ca^2+^ is coordinated by four surrounding residues (β_1_**-**Trp^45^, α_2_**-**Trp^46^/Asp^49^/Ile^51^) and two water molecules (not shown) (PDB: 5Y5S).
